# Redox‐Responsive Dendrimer Nanogels Enable Ultrasound‐Enhanced Chemoimmunotherapy of Pancreatic Cancer via Endoplasmic Reticulum Stress Amplification and Macrophage Polarization

**DOI:** 10.1002/advs.202301759

**Published:** 2023-06-23

**Authors:** Guizhi Zhang, Mengsi Zhan, Changchang Zhang, Zhiqiang Wang, Huxiao Sun, Yuchen Tao, Qiusheng Shi, Meijuan He, Han Wang, João Rodrigues, Mingwu Shen, Xiangyang Shi

**Affiliations:** ^1^ State Key Laboratory for Modification of Chemical Fibers and Polymer Materials Shanghai Engineering Research Center of Nano‐Biomaterials and Regenerative Medicine College of Biological Science and Medical Engineering Donghua University Shanghai 201620 China; ^2^ Department of Ultrasound Shanghai General Hospital Shanghai Jiao Tong University School of Medicine Shanghai 200080 China; ^3^ Department of Radiology Shanghai General Hospital Shanghai Jiao Tong University School of Medicine Shanghai 200080 China; ^4^ CQM – Centro de Química da Madeira MMRG Universidade da Madeira Campus Universitário da Penteada Funchal 9020‐105 Portugal

**Keywords:** chemoimmunotherapy, dendrimer nanogels, immunogenic cell death, redox‐responsiveness, ultrasound‐targeted microbubble destruction

## Abstract

Developing a multifunctional nanoplatform to achieve efficient theranostics of tumors through multi‐pronged strategies remains to be challenging. Here, the design of the intelligent redox‐responsive generation 3 (G3) poly(amidoamine) dendrimer nanogels (NGs) loaded with gold nanoparticles (Au NPs) and chemotherapeutic drug toyocamycin (Au/Toy@G3 NGs) for ultrasound‐enhanced cancer theranostics is showcased. The constructed hybrid NGs with a size of 193 nm possess good colloidal stability under physiological conditions, and can be dissociated to release Au NPs and Toy in the reductive glutathione‐rich tumor microenvironment (TME). The released Toy can promote the apoptosis of cancer cells through endoplasmic reticulum stress amplification and cause immunogenic cell death to maturate dendritic cells. The loaded Au NPs can induce the conversion of tumor‐associated macrophages from M2‐type to antitumor M1‐type to remodulate the immunosuppressive TME. Combined with antibody‐mediated immune checkpoint blockade, effective chemoimmunotherapy of a pancreatic tumor mouse model can be realized, and the chemoimmunotherapy effect can be further ultrasound enhanced due to the sonoporation‐improved tumor permeability of NGs. The developed Au/Toy@G3 NGs also enable Au‐mediated computed tomography imaging of tumors. The constructed responsive dendrimeric NGs tackle tumors through a multi‐pronged chemoimmunotherapy strategy targeting both cancer cells and immune cells, which hold a promising potential for clinical translations.

## Graphical abstract

Intelligent redox‐responsive dendrimer nanogels (NGs) loaded with both gold nanoparticles (Au NPs) and chemotherapeutic drug toyocamycin (Toy) have been developed. The hybrid NGs can be dissociated to release Au NPs and Toy in the reductive tumor microenvironment for ultrasound‐enhanced chemoimmunotherapy of a pancreatic tumor model via Toy‐mediated endoplasmic reticulum stress amplification, Au NP‐mediated macrophage polarization, and antibody‐mediated immune checkpoint blockade.

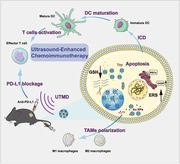

## 1 Introduction

Cancer, a malignant disease caused by the abnormal proliferation of cells in the organism, has become a worldwide health problem due to its low cure and high recurrence rates.^[^ ^1^ ^]^ In the biomedical field, chemotherapy remains the mainstay treatment for many solid malignancies despite the continuous development of new treatment modalities.^[^ ^2^ ^]^ It is worth noting that chemotherapeutic drugs often damage the normal tissues to varying degrees during the treatment process, causing a series of host reactions and forming a microenvironment more suitable for malignant tumor metastasis.^[^ ^3^ ^]^ In addition, the immunosuppressive microenvironment of tumors is characterized by low levels of tumor‐infiltrating cytotoxic T cells (CTLs) and high levels of immunosuppressive cells such as regulatory T cells (Tregs) and M2‐type tumor‐associated macrophages (TAMs), greatly limiting the efficacy of chemotherapy.^[^ ^4^ ^]^ Therefore, the dual strategy of selecting appropriate chemotherapeutic drug targets and regulating the immune microenvironment would effectively induce the immune response of the body with desired therapeutic outcome.^[^ ^5^ ^]^

Among many chemotherapeutic drugs, organelle‐targeted tumor treatment has been prevailing. In particular, the treatment of endoplasmic reticulum (ER) as a pharmacological target is promising.^[^ ^6^ ^]^ It is well known that the ER is a major site for protein folding and trafficking, involved in a variety of main cellular functions.^[^ ^7^ ^]^ Excessive proliferation of tumor cells leads to an increase of misfolded and unfolded proteins, which triggers ER stress (ERS).^[^ ^8^ ^]^ To solve the issue, tumor cells initiate an adaptive response process to restore cellular homeostasis, known as unfolded protein response (UPR).^[^ ^9^ ^]^ The UPR mainly involves three signaling pathways, among which IRE1*α* is the most conserved evolutionary branch of the UPR. The activated IRE1*α* catalyzes the splicing of X‐Box binding protein 1 (XBP1) mRNA to form XBP1s, which can regulate the expression of related genes to restore homeostasis and survival of cancer cells.^[^ ^10^ ^]^ Toyocamycin (Toy), a purine nucleoside analog, has been proven to be an ERS inhibitor to promote tumor cell apoptosis by blocking the IRE1*α*‐XBP1 pathway.^[^ ^11^ ^]^ However, the clinical application of free Toy faces great challenges due to its rapid metabolization and low bioavailability.^[^ ^12^ ^]^

Beyond the chemotherapy effect targeting ERS, persistent ERS can also cause immunogenic cancer cell death (ICD), which drives tumor cells to secrete damage‐associated molecular patterns (DAMPs), including calreticulin (CRT), adenosine triphosphate (ATP) and high mobility group box 1 (HMGB‐1) as danger signals to mature dendritic cells (DCs), activating CTLs in tumors and ultimately inducing a highly effective antitumor immune response.^[^ ^13^ ^]^ However, some chemotherapy drugs can not only induce ICD but also increase the expression of immune checkpoint ligands, leading to the immune escape of tumor cells.^[^ ^14^ ^]^ Immune checkpoint blockade (ICB) therapy has been developed to enhance tumor antigen recognition and cytotoxic activity by reactivating exhausted T cells with the utilization of antibodies such as programmed cell death ligand 1 (PD‐L1).^[^ ^15^ ^]^

For effective cancer therapy, it is necessary to develop nanoplatforms integrating both diagnosis and treatment elements to achieve efficient theranostics. Gold nanoparticles (Au NPs) have attracted much attention for computed tomography (CT) imaging due to the high atomic number of Au, the high X‐ray absorption coefficient, and good biocompatibility.^[^ ^16^ ^]^ Meanwhile, Au NPs‐based nanomaterials could induce the macrophage polarization from pro‐tumor M2‐type to antitumor M1‐type to achieve efficient treatment under CT imaging guidance.^[^ ^17^ ^]^ Therefore, it would be ideal to construct a suitable nanoplatform to combine chemotherapy drugs Toy and Au NPs to exert the chemoimmunotherapy and CT imaging for effective theranostics of tumors.

The development of nanomedicine platforms provides the possibility to solve the shortcomings of traditional drugs.^[^ ^18^ ^]^ In particular, the construction of an intelligent stimuli‐responsive nanosystem is of great significance to realize the accurate and efficient release of chemotherapeutic drugs and imaging agents at the tumor site.^[^ ^19^ ^]^ Nanogels (NGs) possess a three‐dimensional network and can be used as a systemic drug delivery carrier with high hydration and contraction‐expansion properties, which can improve the drug‐loading capacity.^[^ ^20^ ^]^ Amine‐terminated generation 3 poly(amidoamine) (PAMAM) dendrimers (G3.NH_2_) have been used to synthesize NGs due to their highly branched structure, biocompatibility, and surface functional groups.^[^ ^21^ ^]^ On one hand, NGs can prolong the blood circulation of the drug and reduce the risk of toxicity in normal tissues through the enhanced permeability and retention (EPR)‐based passive targeting effect;^[^ ^22^ ^]^ on the other hand, NGs can be designed to possess tumor microenvironment (TME) responsiveness (e.g., redox) to realize the responsive release of chemotherapy drugs, thus improving curative effect and reducing side effects of drugs.^[^ ^23^ ^]^

It is well known that pancreatic cancer has limited delivery of chemotherapy drugs due to their rich matrix, fewer blood vessels, and lack of blood supply barrier.^[^ ^24^ ^]^ Beyond the passive targeting through the ERP effect,^[^ ^25^ ^]^ ultrasound‐targeted microbubble destruction (UTMD) has been proven to increase blood perfusion for improved tumor drug delivery through the ultrasonic cavitation effect.^[^ ^26^ ^]^ Previously, we have demonstrated that a dendrimeric nanoplatform combined with UTMD can remarkably improve the delivery efficiency of gemcitabine and microRNA inhibitor to tumors.^[^ ^27^ ^]^

**Scheme 1:**
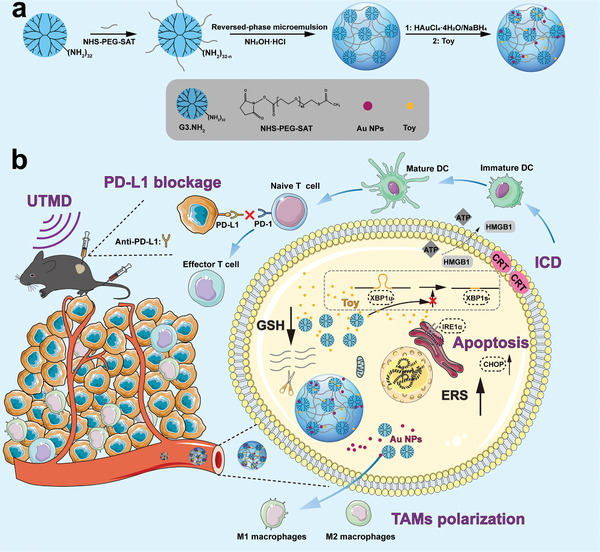
Construction of Au/Toy@G3 NGs (a) for UTMD‐enhanced chemoimmunotherapy and CT imaging of pancreatic tumor in combination with anti‐PD‐L1‐mediated ICB (b).

Here, we report a UTMD‐facilitated synergistic therapeutic nanoplatform based on redox‐responsive NGs loaded with both Au NPs and Toy for combined chemoimmunotherapy (**Scheme** **1**). Firstly, G3.NH_2_ dendrimers were modified with NHS‐PEG‐SAT to form G3‐PEG‐SAT as a macromonomer, which was subsequently self‐crosslinked to generate redox‐responsive G3 NGs containing disulfide bonds via a reverse microemulsion method. The G3 NGs were then adopted as a nanoreactor to in situ immobilize Au NPs and physically load Toy to form the Au/Toy@G3 NGs. We systematically characterized the Au/Toy@G3 NGs including morphology, size, composition, stability, and drug release ability, and evaluated their UTMD‐promoted cytotoxicity, cellular uptake, cell apoptosis, effects on ERS amplification, macrophage polarization, and ICD induction in vitro. Finally, we explored their potential to be used for CT imaging and UTMD‐facilitated pancreatic tumor therapy in vivo, as well as their effect on tumor suppression in combination with PD‐L1 antibody (Anti‐PD‐L1). The designed Au/Toy@G3 NGs possess significant features: 1) The formed hybrid NGs can be easily prepared and responsively release Au NPs and Toy in the TME to improve their bioavailability and reduce side effects; 2) Toy‐induced chemotherapy can trigger the ERS‐mediated tumor cell apoptosis and ICD for DCs maturation to stimulate antitumor immune response; 3) Au NPs can convert TAMs from M2‐type to M1‐type to remodulate the immune suppressive TME and also enable CT imaging; 4) The adopted UTMD technology promotes the accumulation of hybrid NGs in tumor site for improved theranostic efficacy; 5) Further combination with Anti‐PD‐L1 enables significantly improved antitumor immune responses to enhance the chemoimmunotherapy effect.

## 2 Results and Discussion

### 2.1 Synthesis and Characterization of Au/Toy@G3 NGs

The redox‐responsive G3 PAMAM dendrimer nanogels (G3 NGs) were designed for loading Au NPs and chemotherapeutic drug Toy. The synthetic route of Au/Toy@G3 NGs is displayed in Scheme 1. Firstly, NHS‐PEG‐SAT was modified on the surface of G3.NH_2_ by *N*, *N*‐diisopropylethylamine (DIEA)‐catalyzed acylation reaction between the amine groups of G3.NH_2_ and NHS‐PEG‐SAT to form G3‐PEG‐SAT dendrimers. ^1^H NMR was adopted to confirm the structure of G3‐PEG‐SAT (Figure S1, Supporting Information). The peaks of methylene protons in G3 dendrimer are located at 2.2–3.4 ppm, and the PEG proton peaks of NHS‐PEG‐SAT are at 3.7–4.0 ppm. The number of NHS‐PEG‐SAT modified on each G3.NH_2_ was measured to be 6 by ^1^H NMR integration. The resulting G3‐PEG‐SAT dendrimers were used as the macromonomers to synthesize redox‐responsive G3 NGs with disulfide bonds under the action of hydroxylamine hydrochloride to oxidize SAT groups via a reverse microemulsion method.

After that, the prepared G3 NGs were used as a nanoreactor to form Au@G3 NGs by entrapping Au NPs in situ through a rapid sodium borohydride reduction method. The Au content was measured to be 8.4 wt% in Au@G3 NGs by inductively coupled plasma‐optical emission spectrometry (ICP‐OES). Next, Toy was loaded into the Au@G3 NGs by physical encapsulation (Au/Toy@G3 NGs). We first prepared the Toy‐loaded Au@G3 NGs with different G3.NH_2_/Toy mass feeding ratios (1:0.1, 1:0.25, and 1:0.5, respectively) to optimize the Toy encapsulation efficiency (EE) and loading capacity (LC). The loading of Toy in the Au/Toy@G3 NGs was analyzed by UV–vis spectroscopy using Toy absorbance at 280 nm per concentration calibration curve (Figure S2, Supporting Information). As shown in Table S1, Supporting Information, the mass ratio of G3.NH_2_/Toy was optimized to be 1: 0.25 to have the balanced EE (57.0%) and LC (12.5%) of Toy.

**Figure 1:**
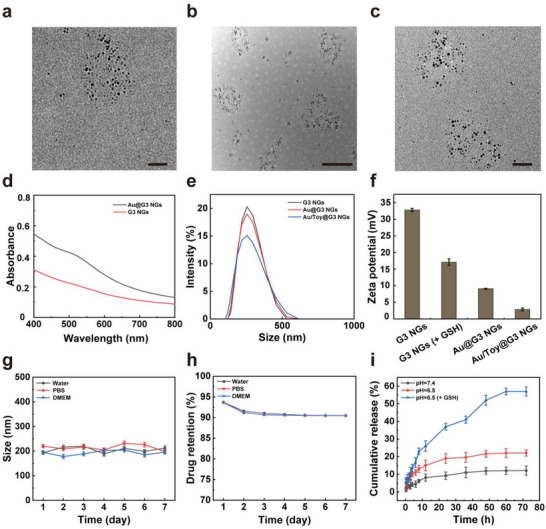
TEM images of a) Au@G3 NGs (scale bar is 50 nm) and b,c) Au/Toy@G3 NGs (scale bar represents 200 nm and 50 nm, respectively). d) UV–vis absorption spectra of G3 NGs and Au@G3 NGs. e) The hydrodynamic size distribution of G3 NGs, Au@G3 NGs, and Au/Toy@G3 NGs. f) Zeta potentials of G3 NGs with or without GSH (10 mm), Au@G3 NGs, and Au/Toy@G3 NGs. g) The hydrodynamic size of the Au/Toy@G3 NGs dispersed in water, PBS, or DMEM (containing 10% FBS) for one week at room temperature. h) Toy retention in the Au/Toy@G3 NGs after the NGs were dispersed in water, PBS or DMEM (containing 10% FBS) for one week at room temperature. i) The cumulative release of Toy from the Au/Toy@G3 NGs at pH 7.4 or pH 6.5 in the presence or absence of GSH (10 mm). For f)‐i), data are presented as mean ± SD, *n* = 3 for each sample.

The synthesized Au@G3 NGs were observed by transmission electron microscopy (TEM) to show a spherical shape and uniform size distribution of entrapped Au NPs (**Figure** **1**a and Figure S3, Supporting Information). The average diameters of the whole NGs and single Au NPs were measured to be about 150 nm and 5 nm, respectively. After Toy encapsulation, the spherical morphology and size of the Au/Toy@G3 NGs do not seem to have appreciable changes (Figure 1b,c). UV–vis spectroscopy reveals a characteristic peak of Au@G3 NGs at 520 mm that can be attributed to the surface plasmon resonance peak of Au NPs, verifying the successful entrapment of Au NPs in the G3 NGs (Figure 1d).

The hydrodynamic size and zeta potential measurements were also performed to characterize the synthesized NGs. As displayed in Table S2, Supporting Information and Figure S4, Supporting Information, G3 NGs display a hydrodynamic size of 237.9 ± 6.4 nm, while the size decreases to 125.8 ± 2.9 nm in the presence of glutathione (GSH, 10 mm), demonstrating the redox‐responsive disintegration of G3 NGs through disulfide bond fracture. The hydrodynamic sizes of Au@G3 NGs and Au/Toy@G3 NGs decrease to 204.7 ± 3.1 and 193.0 ± 4.1 nm (Table S2, Supporting Information and Figure 1e), respectively, which may be due to the loading of Au NPs and Toy that tightens the NGs network to have shrunken volumes. Interestingly, the size of the Au@G3 NGs or Au/Toy@G3 NGs measured by dynamic light scattering (DLS) is larger than those tested by TEM (Figure 1a–c and Figure S3, Supporting Information). This should be because DLS evaluates the hydrodynamic size of NGs with the expanded state in an aqueous solution, while TEM only measures the particle size in a dry state. After in situ entrapment of Au NPs and encapsulation of Toy in G3 NGs, the zeta potentials of Au@G3 NGs and Au/Toy@G3 NGs decrease to 9.1 ± 0.1 and 2.9 ± 0.4 mV, respectively, which is in accordance with the literature,^[^ ^20a^ ^]^ indicating the successful incorporation of Au NPs and Toy (Figure 1f). It is noted that the polydispersity index of G3 NGs, Au@G3 NGs, and Au/Toy@G3 NGs are all less than 0.4, suggesting the good dispersion and uniformity of prepared NGs in water.

The stability of the Au@G3 NGs and Au/Toy@G3 NGs was assessed by checking the changes of their hydrodynamic sizes in water, phosphate buffered saline (PBS) or cell culture medium (DMEM with 10% fetal bovine serum (FBS)) within one week (Figure S5, Supporting Information and Figure 1g). The hydrodynamic diameters of both Au@G3 and Au/Toy@G3 NGs have no significant changes in various media at room temperature, indicating their acceptable colloidal stability. To further verify the stability of Toy in the Au/Toy@G3 NGs, we measured the Toy concentration in the supernatant after the NG suspensions in water, PBS or cell culture medium were centrifuged. Clearly, the retention of Toy in the Au/Toy@G3 NGs remains up to 90% for 7 days, indicating the good stability of Toy remained in the NGs (Figure 1h).

The redox‐responsive release of Toy from the Au/Toy@G3 NGs under different conditions (pH 7.4 or pH 6.5 in the presence and absence of 10 mm GSH) within 72 h was investigated. As revealed in Figure 1i, the release of Toy increases from 12.1% at pH 7.4 to 22.1% at pH 6.5, which is mainly attributed to the more swollen network structure of the Au/Toy@G3 NGs with protonated dendrimer amines under acidic conditions.^[^ ^21b^ ^]^ The Toy release reaches as high as 57.0% in the presence of GSH at pH 6.5, which is much higher than in the absence of GSH (22.1%), indicating the redox‐responsive release of Toy due to the GSH‐mediated decomposition of Au/Toy@G3 NGs. Hence, the constructed redox‐responsive Au/Toy@G3 NGs can be stable in blood circulation to prevent early leakage of Toy, and respond to TME with a high GSH level for fast Toy release in tumors.

### 2.2 Cytotoxicity and Cellular Uptake Assays

**Figure 2:**
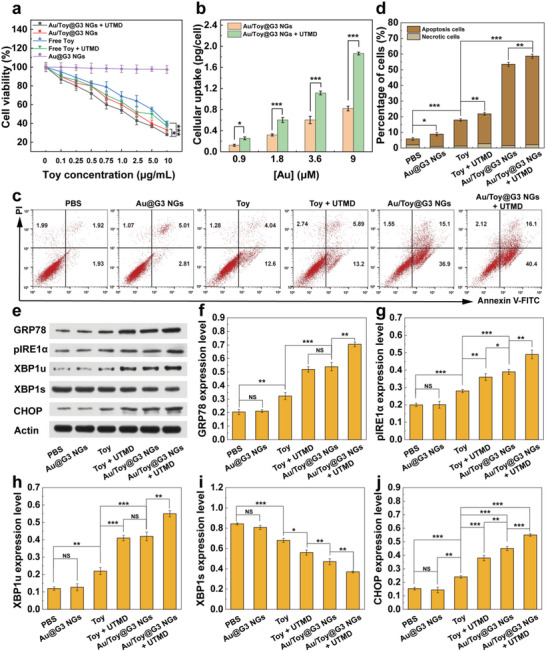
a) Viability of the Pan02 cells treated with Au@G3 NGs, Toy, Toy + UTMD, Au/Toy@G3 NGs, and Au/Toy@G3 NGs + UTMD at diverse Toy concentrations for 24 h (*n* = 6). b) Au uptake in Pan02 cells after cells were treated with Au/Toy@G3 NGs in the presence or absence of UTMD at different Au concentrations for 6 h (*n* = 3). c) Flow cytometry analysis of Pan02 cell apoptosis after various treatments for 12 h. d) Quantitative analysis of apoptosis rate of Pan02 cells after different treatments for 12 h (*n* = 3). e) WB assay of ERS‐related proteins in Pan02 cells after they were differently treated for 24 h. The quantification of the expression levels of f) GRP78, g) pIRE1*α*, h) XBP1u, i) XBP1s, and j) CHOP protein relative to *β*‐actin after different treatments for 24 h, respectively (*n* = 3). In parts a),b), d), and f)–j), * is for *p* < 0.05, ** is for *p* < 0.01, and *** is for *p* < 0.001, respectively (data are presented as mean ± SD).

Subsequently, we examined the anticancer activity of Au@G3 NGs, Toy, Toy + UTMD, Au/Toy@G3 NGs, and Au/Toy@G3 NGs + UTMD to Pan02 cells possessing a high level of GSH by cell counting kit‐8 (CCK‐8) assay (**Figure** **2**a). The cell viability remains 97.4% in the given concentration range for the Au@G3 NGs group, suggesting that the drug‐free Au@G3 NGs have almost no cytotoxicity to Pan02 cells. Meanwhile, free Toy reduces the viability of Pan02 cells in a dose‐dependent manner, while a higher inhibitory efficacy is obtained in the treatment group of Toy + UTMD, likely due to the UTMD‐mediated sonoporation effect. Clearly, the Au/Toy@G3 NGs + UTMD display a higher cytotoxicity than the Au/Toy@G3 NGs without UTMD (*p* < 0.05) at the same Toy concentration (10 µg mL^−1^), which should be ascribed to the improved cellular uptake of the NGs with the assistance of UTMD. The half‐maximal inhibitory concentrations (IC_50s_) of Toy were calculated to assess the antiproliferation effect of Pan02 cells (Table S3, Supporting Information). The Au/Toy@G3 NGs + UTMD groups display the lowest IC_50s_ (1.59 µg mL^−1^) among all groups, which should result from the sustained Toy release in Au/Toy@G3 NGs and the augmented cellular uptake of Au/Toy@G3 NGs under UTMD.

Next, we investigated the cellular uptake of Au contents in Pan02 cells after cells were incubated with Au/Toy@G3 NGs in the presence or absence of UTMD at various Au concentrations. ICP‐OES data (Figure 2b) reveal that both groups exhibit a concentration‐dependent Au uptake. Cells treated with the Au/Toy@G3 NGs + UTMD take up more Au than those treated with the Au/Toy@G3 NGs without UTMD at the same Au concentrations (1.8 µm or above, *p* < 0.001). This indicates that the UTMD treatment promotes the cellular uptake of NGs, in agreement with the literature.^[^ ^19^ ^]^

### 2.3 Cancer Cell Apoptosis In Vitro

The cell apoptosis induced by chemotherapy was investigated using flow cytometry (Figure 2c,d). There are almost no apoptotic cells detected in the group of PBS and Au@G3 NGs, while the increased proportions of apoptotic and necrotic cells are detected in the Toy‐related groups. Importantly, the apoptosis rate significantly increases in the group of Au/Toy@G3 NGs + UTMD (56.5%), which is higher than the groups of Toy (16.64%, *p* < 0.001) and Au/Toy@G3 NGs (52.0%, *p* < 0.01), revealing the sustained Toy release from the Au/Toy@G3 NGs can promote cell apoptosis with the assistance of UTMD treatment. The treatment of Au/Toy@G3 NGs + UTMD achieves the highest cell apoptosis rate among all groups, amenable for further biomedical applications.

### 2.4 Amplification of ERS In Vitro

Toy is known to aggravate the ERS process for anticancer applications. RT‐PCR assay was carried out to detect the changes of corresponding mRNA levels in Pan02 cells after different treatments. GRP78, as an ERS marker, significantly increases in cells at the mRNA level to repair protein folding problems after treatment with Toy‐related materials, and the treatment of Au/Toy@G3 NGs + UTMD achieves the highest GRP78 mRNA level due to the Toy‐mediated inhibition of persistent ERS adaptation and UTMD‐enhanced cellular uptake of the NGs (Figure S6a, Supporting Information). XBP1u is an uncleaved XBP1 precursor that can be cleaved into XBP1s to regulate cell survival and metabolism during ERS. As can be seen in Figure S6b,c, Supporting Information, the expression of XBP1u mRNA in cells increases in all the Toy‐related groups, and the treatment of Au/Toy@G3 NGs + UTMD leads to the highest XBP1u mRNA among all groups, about 1.26 and 1.09 times higher than that treated with Toy + UTMD (*p* < 0.001) and Au/Toy@G3 NGs (*p* < 0.05), respectively. In contrast, the expression of XBP1s mRNA decreases in Pan02 cells for the Toy‐related groups, and the treatment of Au/Toy@G3 NGs + UTMD results in the lowest XBP1s mRNA expression level among all groups (*p* < 0.01). It can be seen that the Toy‐related groups have decreased cleavage of XBP1u to XBP1s and reduced adaptability of cells to ERS, thus leading to the apoptosis of tumor cells and inhibition of tumor progression. Meanwhile, as a marker of ERS‐induced apoptosis, the expression level of CHOP was also evaluated and its increased expression promotes cell apoptosis and death (Figure S6d, Supporting Information). The Au/Toy@G3 NGs + UTMD group displays the highest CHOP mRNA expression among all groups (*p* < 0.01), confirming the cell apoptosis through ERS amplification, which is consistent with the cancer cell apoptosis evaluations in vitro.

Western blot (WB) assay was also adopted to detect the expression level of ERS‐related proteins in Pan02 cells after different treatments (Figure 2e–j). The expression levels of GRP78, XBP1u, and CHOP are higher in the Au/Toy@G3 NGs + UTMD group, while the expression level of XBP1s is lower in the same group than in the other groups. This is consistent with the RT‐PCR results (Figure S6, Supporting Information). Further, the expression of pIRE1*α* was also investigated since it serves as an important component of the IRE1*α*‐XBP1 pathway in the ERS process. The treatment of Au/Toy@G3 NGs + UTMD increases the pIRE1*α* expression at the highest level among all groups (*p* < 0.01) because the amplified ERS can enhance the expression of pIRE1*α* in Pan02 cells to alleviate ERS. However, all Toy‐related groups only block the activity of the endonuclease of pIRE1*α* to prevent the splicing of XBP1u to XBP1s without the ability to inhibit pIRE1*α* expression. As described above, the anticancer activity induced by Au/Toy@G3 NGs + UTMD can promote cancer cell death through enhanced ERS‐mediated apoptosis and suppressed ERS adaptive regulation due to the improved bioavailability of the Toy through sonoporation‐promoted drug delivery.

### 2.5 Effect of Au/Toy@G3 NGs on Macrophage Polarization

**Figure 3:**
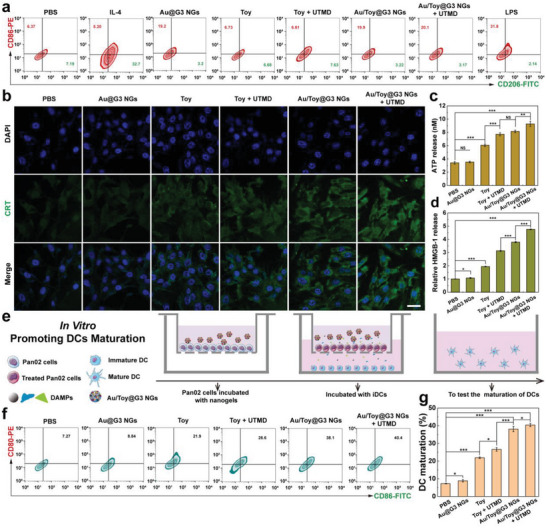
a) Flow cytometry analysis of macrophage polarization after various treatments for 24 h. LPS‐treated RAW264.7 cells were set as the positive control. b) CLSM observation of CRT exposure on the surface of Pan02 cell after various treatments for 24 h (scale bar: 40 µm). c) ATP secretion and d) relative HMGB‐1 release from Pan02 cells after different treatments for 24 h. e) Schematic illustration of the co‐culture transwell system of Pan02 cells treated with different formulations and iDCs. f) Flow cytometry analysis of DCs maturation through detection of CD80‐PE and CD86‐FITC markers. g) Quantification of mature DCs after co‐culture with ICD Pan02 cells after different treatments. In parts c), d), and g), * is for *p* < 0.05, ** is for *p* < 0.01, and *** is for *p* < 0.001, respectively (*n* = 3, data are presented as mean ± SD).

To determine whether the Au/Toy@G3 NGs could induce macrophage polarization in TME, flow cytometry was used to quantify the expression of CD86 (a marker of M1‐type) and CD206 (a marker of M2‐type) in RAW264.7 cells (**Figure** **3**a). We first prepared M2‐type macrophages using IL‐4 (50 ng mL^−1^) as a stimulator except for the PBS group, and then treated them with different formulations. LPS (2 µg mL^−1^) was selected as a positive control. Clearly, Au NPs‐containing groups increase the expression level of CD86 and decrease the CD206 expression as compared to the negative control IL‐4 group, demonstrating the strong ability of Au NPs to convert macrophages from M2‐type to M1‐type. It is noted that the percentages of CD86‐positive cells (19.2% versus 19.9%) and CD206‐positive cells (3.20% versus 3.22%) are almost the same for the Au@G3 NGs and Au/Toy@G3 NGs groups, while the Au/Toy@G3 NGs + UTMD group displays the highest proportion of CD86 (20.1%) and the lowest proportion of CD206 (3.17%) among all groups except for LPS. In addition, the ratio of M1 to M2 was investigated to further check the macrophage polarization promoted by Au/Toy@G3 NGs + UTMD. The ratio of M1/M2 for the group of Au/Toy@G3 NGs + UTMD is 39.87 and 7.32 times higher than for the groups of IL‐4 and Toy + UTMD (Figure S7, Supporting Information), respectively.

As a key cytokine to promote cancer cell death, the content of TNF‐*α* secreted in the culture medium of macrophages after various treatments was then evaluated by ELISA (Figure S8, Supporting Information). The TNF‐*α* level in the Au/Toy@G3 NGs + UTMD group (547.0 pg mL^−1^) is much higher than in the groups of Au/Toy@G3 NGs (457.2 pg mL^−1^, *p* < 0.001) and Toy + UTMD (371.1 pg mL^−1^, *p* < 0.001), indicating the successful M1 polarization of the macrophages for increased secretion of TNF‐*α* to exert effective anticancer therapy.

### 2.6 ICD Effect of Au/Toy@G3 NGs In Vitro

To test the ability of Au/Toy@G3 NGs to induce ICD of Pan02 cells, the changes of representative DAMPs (CRT, ATP, and HMGB‐1) were examined after cells were treated with different materials. The CRT expression on the surface of Pan02 cells was evaluated by immunofluorescence staining (Figure 3b and Figure S9, Supporting Information). Compared with the PBS group, the green fluorescence of CRT on the cell surface significantly increases in the Toy and Toy‐containing groups, which suggests an obvious ICD effect induced by Toy‐mediated chemotherapy due to its direct action on ERS. Further, the additional UTMD treatment promotes the expression of CRT in the Au/Toy@G3 NGs + UTMD group, which achieves the strongest CRT fluorescence signal, successfully promoting the transfer of CRT from endoplasmic to the cell surface. Similarly, the content of ATP secreted in the culture medium of cells treated by the Au/Toy@G3 NGs + UTMD reaches the highest level among all groups (*p* < 0.01, Figure 3c), suggesting the enhanced ICD effect through UTMD‐facilitated improved chemotherapy of the hybrid NGs. As shown in Figure 3d, all Toy‐related groups could promote the release of HMGB‐1 to some extent through apoptosis of Pan02 cells, and the most significant release can be achieved in the group of Au/Toy@G3 NGs + UTMD, which is around 4.8 times higher than in the PBS group (*p* < 0.001).

Since DCs are one of the most typical antigen‐presenting cells (APCs), recognizing the DAMPs released by ICD cancer cells, we then checked ICD‐mediated DCs maturation for activated CTLs to induce effective immunotherapy. In this study, we explored the changes in expression levels of CD80/CD86 co‐stimulatory factors and MHC‐I antigen presentation molecules of DCs to validate their maturation status. A transwell system was built up to verify the maturation of DCs induced by co‐culture of ICD Pan02 cells after different treatments with immature DCs (Figure 3e). It can be seen in Figure 3f,g, compared with the PBS group (7.27%), the maturity of DCs (CD80^+^CD86^+^ cells) in the Au/Toy@G3 NGs group reaches up to 38.1%, much higher than the Au @G3 NGs group without Toy. With the assistance of UTMD technology, the maturity of DCs further increases and reaches the highest among all groups (40.4%, *p* < 0.05). Meanwhile, as shown in Figure S10, Supporting Information, DCs in the group of Au/Toy@G3 NGs + UTMD display the most significant expression of MHC‐I, one important marker of DC‐mediated antigen presentation among all groups (*p* < 0.001). Collectively, these results imply that the Au/Toy@G3 NGs + UTMD can trigger an efficient ICD effect of Pan02 cells to improve the antigen presentation ability of DCs in vitro, which is amenable to further activate the antitumor immune response.

### 2.7 Hemolysis Assay and Pharmacokinetics

The hemocompatibility of the Au/Toy@G3 NGs was assessed before they were used for in vivo studies. As exhibited in Figure S11, Supporting Information, no significant hemolysis (less than 5%) of mouse red blood cells (RBCs) can be observed at a Toy concentration up to 5 µg·mL^−1^, indicating the good blood compatibility of the Au/Toy@G3 NGs. It is also important to examine the pharmacokinetics of Au/Toy@G3 NGs before evaluating their therapeutic activity in vivo. As can be clearly seen in Figure S12, Supporting Information, the half‐decay time (t_1/2_) of the Au/Toy@G3 NGs reaches 3.189 h, which is relatively long and conducive to rendering the NGs with EPR‐based passive accumulation at the tumor site.

### 2.8 In Vivo CT Imaging and Biodistribution

**Figure 4:**
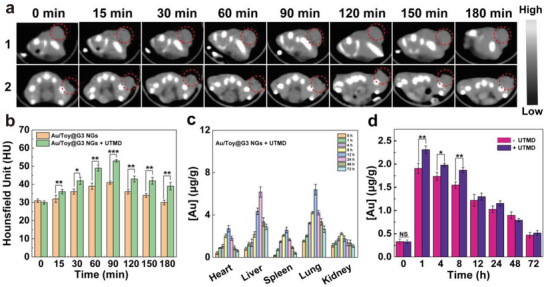
In vivo a) CT images and b) CT values of Pan02 tumor‐bearing mice at different time intervals after intravenous injection of (1) Au/Toy@G3 NGs and (2) Au/Toy@G3 NGs + UTMD ([Au] = 10 mm, 0.1 mL). c) Biodistribution of Au element in the major organs of mice at different time points post intravenous injection of Au/Toy@G3 NGs + UTMD ([Au] = 10 mm, 0.1 mL). d) Tumor uptake of Au at different time points post intravenous injection of Au/Toy@G3 NGs and Au/Toy@G3 NGs + UTMD ([Au] = 10 mm, 0.1 mL), respectively. In parts b) and d), * is for *p* < 0.05, ** is for *p* < 0.01, and *** is for *p* < 0.001, respectively. For b‐d, *n* = 3, data are presented as mean ± SD.

The CT imaging potential of the Au/Toy@G3 NGs was then investigated since Au element possesses a high atomic number and a strong X‐ray attenuation coefficient. As shown in Figure S13, Supporting Information, the Au/Toy@G3 NGs exhibit an Au concentration‐dependent increase in CT value with a slope of 7.74 HU·mM^−1^, suggesting their great potential in CT imaging. Afterward, the tumor CT imaging performance of Au/Toy@G3 NGs in the presence and absence of UTMD was subsequently validated through a Pan02 tumor‐bearing mouse model. Before injection, the CT value in the tumor area is relatively low. However, after intravenous injection of the Au/Toy@G3 NGs or Au/Toy@G3 NGs + UTMD, the CT value gradually increases with time and reaches a peak value at 90 min (**Figure** **4**a,b). At 90 min post‐injection, the CT value at the tumor site in the Au/Toy@G3 NGs + UTMD group is much higher than in the Au/Toy@G3 NGs group (*p* < 0.001), showing a greater CT imaging performance of Au/Toy@G3 NGs + UTMD. This should be due to the created sonoporation effect by UTMD treatment to render the Au/Toy@G3 NGs with deep tumor penetration and accumulation, in agreement with the literature.^[^ ^28^ ^]^

To further study the metabolic behavior of mice after intravenous injection of the Au/Toy@G3 NGs with or without UTMD, the biodistribution of Au element in different organs including heart, liver, spleen, lung, kidney, and tumor was quantitatively determined by ICP‐OES at different time points post‐injection (Figure 4c,d and Figure S14, Supporting Information). Both Au/Toy@G3 NGs and Au/Toy@G3 NGs + UTMD treatments show increased Au accumulation in all organs and tumor at the early stage, and then declines with the extension of time post‐injection, suggesting that the Au/Toy@G3 NGs can be regularly metabolized. Interestingly, the metabolic process of Au/Toy@G3 NGs in the presence of UTMD is similar to that in the absence of UTMD and displays an enhanced Au content at the tumor site after UTMD treatment, in agreement with the CT imaging data. However, it should be noted that there was also a slight increase in Au content observed at the lung site. These results indicate that the application of UTMD does not change the biodistribution behavior of Au/Toy@G3 NGs except the lung in vivo, but significantly promotes the accumulation of NGs at tumor site.

### 2.9 In Vivo Therapeutic Efficacy Evaluation

**Figure 5:**
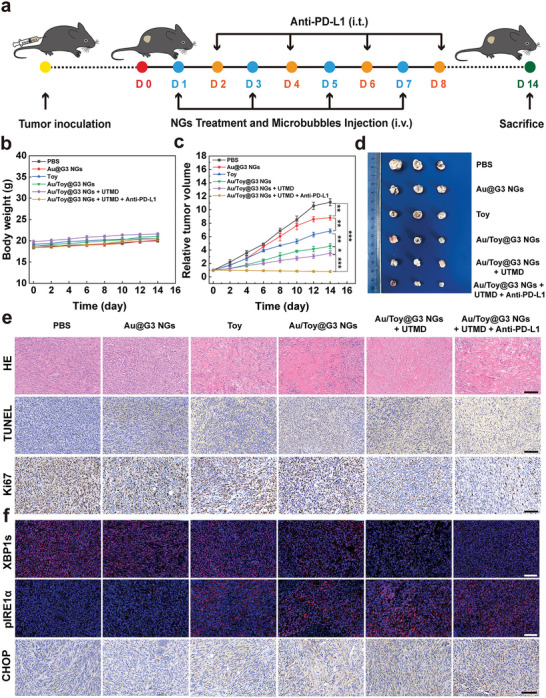
a) Timeline of the in vivo chemotherapy in combination with ICB treatment. b) Body weights and c) relative tumor volumes of Pan02 tumor‐bearing mice after different treatments within 14 days. d) Representative tumor photographs of mice in each group after different treatments (*n* = 3). e) H&E, TUNEL, and Ki67 staining of tumor tissue after the mice were treated for 14 days in different groups. The scale bar for each panel represents 100 µm in H&E, TUNEL and Ki67 staining images. f) Immunofluorescence staining of XBP1s and pIRE1*α* and immunohistochemical analysis of CHOP in tumor slices after the mice were differently treated. The scale bar for each panel represents 100 µm in XBP1s, pIRE1*α* and CHOP staining images. In part c, * is for *p* < 0.05, ** is for *p* < 0.01, and *** is for *p* < 0.001, respectively. For b) and c), data are presented as mean ± SD, *n* = 5 in each group.

We further studied the therapeutic effect of the Au/Toy@G3 NGs under UTMD in vivo. Considering that the Au/Toy@G3 NG‐mediated chemotherapy not only produces ICD effect to activate T cells to attack tumor cells, but also may cause the overexpression of PD‐L1 in tumor cells to intensify the immune escape.^[^ ^1^ ^]^ We firstly evaluated the expression of PD‐L1 on Pan02 cells after different treatments in vitro. As shown in Figure S15, Supporting Information, the PD‐L1 expression is more significantly upregulated on cells treated with Au/Toy@G3 NGs + UTMD than on cells in other groups (*p* < 0.05), indicating that the UTMD‐enhanced chemotherapy could potentially trigger the self‐defense mechanism of cancer cells and render them immunosuppressive. So we combined the treatment of Au/Toy@G3 NGs under UTMD with anti‐PD‐L1‐mediated ICB to enhance the antitumor efficacy and immune response. The established Pan02 tumor‐bearing mice were assigned to six groups and treated according to the schedule shown in **Figure** **5**a. The tumor‐bearing mice after different treatments all shows slight weight gain within 14 days, indicating that the prepared NGs with or without different combinations do not cause serious side effects in vivo (Figure 5b). The changes in tumor volume in different groups were recorded (Figure S16, Supporting Information). The antitumor effect was also evaluated by monitoring the changes in relative tumor volume and actual tumor size after various treatments (Figure 5c,d). Compared with the PBS group, the tumor growth rate of mice in the Au@G3 NGs group is slowed down to a certain extent (*p* < 0.01), which is likely due to the TAMs polarization from M2‐type to M1‐type induced by Au@G3 NGs. Interestingly, a more effective antitumor effect can be achieved after treatment of Au/Toy@G3 NGs due to the additional Toy‐associated chemotherapy effect through EPR‐based passive targeting of the NGs to the tumors. In addition, the Au/Toy@G3 NGs exhibit a more effective tumor suppressive effect in the presence of UTMD than the Au/Toy@G3 NGs alone (*p* < 0.05), possibly due to the enhanced accumulation and penetration of Au/Toy@G3 NGs through UTMD‐induced sonoporation effect. To further confirm whether the remarkable inhibition of tumor growth by Au/Toy@G3 NGs with the help of UTMD can produce a thermal effect, we performed infrared thermal imaging of mice before and after UTMD treatment. As shown in Figure S17, Supporting Information, no obvious temperature rise can be observed in the tumor site before (0 min) or after UTMD treatment. Therefore, the UTMD treatment based on the selected parameters has no thermal effects to tumor site. More importantly, the combination treatment of Au/Toy@G3 NGs + UTMD + Anti‐PD‐L1 possesses the best tumor inhibition effect among all groups (*p* < 0.001). Further survival analysis showed that 40% of the tumor‐bearing mice in the Au/Toy@G3 NGs + UTMD + Anti‐PD‐L1 group were still alive after 69 days and only 20% mice in the Au/Toy@G3 NGs + UTMD group survived at the same time point, indicating the effective therapeutic effect of chemical immunotherapy combined with ICB therapy. In any case, the mice in the groups of PBS, Au@G3 NGs, Toy, and Au/Toy@G3 NGs all died after 34, 48, 51, and 64 days, respectively (Figure S18, Supporting Information). This suggests that the ICB combined with TAMs‐based immunomodulation and chemotherapy leads to the most effective tumor inhibition among all groups.

At 14 days of the treatment, the tumors were also subjected to B‐mode ultrasound (US) imaging to evaluate the therapeutic effect of combination therapy (Figure S19a,b, Supporting Information). In comparison with other groups, the tumor volume of mice is more significantly reduced in the group of Au/Toy@G3 NGs + UTMD + Anti‐PD‐L1, verifying the most effective therapeutic effect of the combination treatment. Since the intratumoral blood perfusion in the tumor region can be used as a prognostic indicator, contrast‐enhanced ultrasound (CEUS) imaging was executed to examine the tumor vessels. The echo intensity of tumors in different groups quantified (Figure S19c, Supporting Information) reveals that the tumors treated with the Au/Toy@G3 NGs + UTMD + Anti‐PD‐L1 possess the highest intratumoral blood perfusion among all groups (*p* < 0.001), indicating the best tumor prognosis.

The excellent antitumor effect of Au/Toy@G3 NGs + UTMD + Anti‐PD‐L1 was next confirmed by hematoxylin and eosin (H&E), TdT‐mediated dUTP Nick‐End Labeling (TUNEL), and Ki67 staining of tumor slices (Figure 5e). Consistent with the above tumor inhibition evaluation, it is clear that the Au/Toy@G3 NGs + UTMD + Anti‐PD‐L1 group possesses the most significant tumor cell necrosis, apoptosis and proliferation inhibition among all groups. Quantitative analysis of Ki67 staining (Figure S20a, Supporting Information) shows that the tumor cell proliferation rate is in the order of PBS (83.3%) > Au@G3 NGs (61.8%) > Toy (47.6%)> Au/Toy@G3 NGs (40.0%) > Au/Toy@G3 NGs + UTMD (25.3%) > Au/Toy@G3 NGs + UTMD + Anti‐PD‐L1 (10.2%), while the tumor cell apoptosis rate (TUNEL‐positive cells, Figure S20b, Supporting Information) is in an order of PBS (5.6%) < Au@G3 NGs (19.0%) < Toy (31.5%) < Au/Toy@G3 NGs (47.1%) < Au/Toy@G3 NGs + UTMD (63.5%) < Au/Toy@G3 NGs + UTMD + Anti‐PD‐L1 (78.2%).

The antitumor mechanism of the combined treatment was next investigated in vivo by measuring the representative proteins in tumors. The ERS‐related markers of XBP1s and pIRE1*α* were evaluated by immunofluorescence staining, while the CHOP marker was assessed by immunohistochemical staining (Figure 5f). As can be clearly shown in Figure S20c, Supporting Information, the free Toy and Toy‐containing groups highly inhibit the expression of XBP1s to varying degrees, likely due to the Toy‐mediated inhibition of the conversion of XBP1u to XBP1s, in consistence with the RT‐PCR and WB results in vitro (Figure S6, Supporting Information and Figure 2e–j). Owing to the exacerbation of ERS status, pIRE1*α* expression increases significantly in tumors as the red fluorescence becomes stronger in the Toy‐related groups than in the Toy‐free groups (Figure S20d, Supporting Information). Meanwhile, the expression of CHOP also increases in the Toy‐related groups, indicating that tumor cells undergo an ERS‐induced apoptosis. The quantitative analysis of CHOP shows that the highest expression level can be achieved in the groups of Au/Toy@G3 NGs + UTMD and Au/Toy@G3 NGs + UTMD + Anti‐PD‐L1 (Figure S20e, Supporting Information). Overall, the combination treatment of NGs under UTMD with or without Anti‐PD‐L1 can effectively inhibit the IRE1*α*‐XBP1 pathway‐mediated tumor cell survival and promote the ERS‐mediated apoptosis pathway.

### 2.10 Immune Activation and TME Modulation

**Figure 6:**
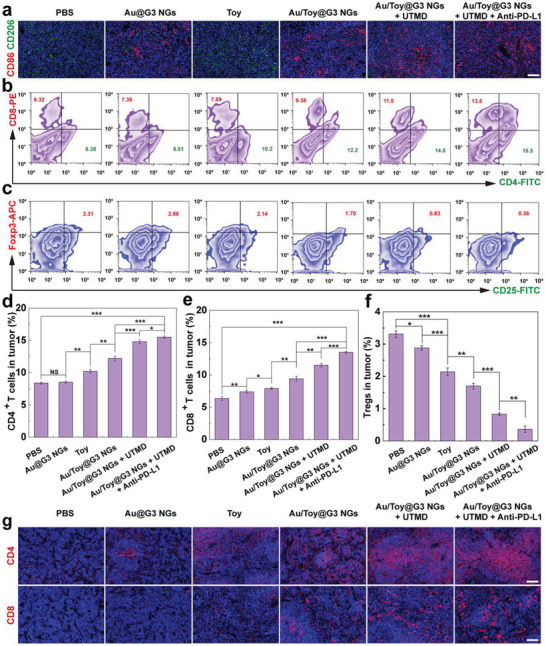
a) The immunofluorescence staining of CD86^+^ and CD206^+^ TAMs in tumors at 14 days post‐treatment in different groups (scale bar = 100 µm for each panel). Representative flow cytometry plots indicating the proportions of b) CD4^+^/CD8^+^ T cells and c) Tregs (Foxp3^+^CD25^+^ T cells gated on CD4^+^ T cells) in tumors after the mice were differently treated for 14 days. Percentages of d) CD4^+^ T cells, e) CD8^+^ T cells, and f) Tregs in tumors after the mice were differently treated for 14 days. g) Immunofluorescence staining of CD4^+^ and CD8^+^ T cells in spleens after the mice were differently treated for 14 days. The scale bar for each panel represents 100 µm. In parts d)–f), * is for *p* < 0.05, ** is for *p* < 0.01, and *** is for *p* < 0.001, respectively (*n* = 3, data are presented as mean ± SD).

Reversing the tumor immune microenvironment by taking down immunosuppressive cells (such as M2‐type TAMs and Tregs) and increasing the supportive immune cells (like CD4^+^ and CD8^+^ T cells) is essential for improved tumor immunotherapy. We next explored the immune activation of TME regulated by the treatment of Au/Toy@G3 NGs + UTMD + Anti‐PD‐L1. To validate the effect of TAMs polarization in tumors after different treatments, the collected tumor sections were stained by CD86 and CD206 antibodies, which represent M1‐type and M2‐type macrophages, respectively (**Figure** **6**a). Compared with the PBS group, the red fluorescence of CD86 in the Au NPs‐containing groups increases, while the green fluorescence of CD206 decreases, indicating that Au NPs could efficiently convert TAMs from M2 to M1‐type, which is consistent with the in vitro results. Notably, the highest CD86 expression level and the lowest CD206 expression level can be achieved in the groups of Au/Toy@G3 NGs + UTMD and Au/Toy@G3 NGs + UTMD + Anti‐PD‐L1 (Figure S21, Supporting Information), indicating that the immunosuppressive TME has been successfully remodulated to be an antitumor one.

Next, the ICD effect induced by Toy‐related groups was validated in vivo. Immunofluorescence staining of CRT expression in tumor cells (Figure S22, Supporting Information) reveals that all Toy‐containing groups have much higher CRT expression than the PBS group, similar to the in vitro studies. The groups of Au/Toy@G3 NGs + UTMD and Au/Toy@G3 NGs + UTMD + Anti‐PD‐L1 show the highest level of green fluorescence and the most significant CRT translocation among all groups (*p* < 0.05), indicating the effective ICD effect induced by the combination therapy in vivo through TAM regulation and chemotherapy. Subsequently, to evaluate the antitumor immunity induced by the Au/Toy@G3 NGs + UTMD + Anti‐PD‐L1, flow cytometry was used to analyze the proportion of tumor‐infiltrating CTLs, including CD4^+^, CD8^+^ T cells, and Tregs (Figure 6b). The proportion of CD4^+^ and CD8^+^ T cells significantly increases in the Toy‐related groups, which should be due to the ICD‐induced effect for DCs maturation to bring an effective antitumor immune response. In addition, the proportion of CD4^+^ and CD8^+^ T cells is the highest under the combined treatment of Au/Toy@G3 NGs + UTMD + Anti‐PD‐L1, about 1.85 and 2.13 times higher than the PBS group (Figure 6d,e), respectively. It is noted that the amount of CD4^+^ T and CD8^+^ T lymphocytes in the Au/Toy@G3 NGs + UTMD + Anti‐PD‐L1 group is much higher than that of Au/Toy@G3 NGs group, which should be due to the UTMD‐promoted chemotherapy and ICB‐mediated immunotherapy (*p* < 0.001).

The tumor‐infiltrating Tregs negatively modulate immunotherapy response, hence reducing the proportion of Tregs in tumors is necessary to warrant successful immunotherapy. As shown in Figures 6c and 6f, the amount of Tregs (Foxp3^+^CD25^+^ T cells gated CD4^+^ T cells) in tumors follows an order of PBS (3.31%) > Au@G3 NGs (2.88%) > free Toy (2.14%) > Au/Toy@G3 NGs (1.70%) > Au/Toy@G3 NGs + UTMD (0.83%) > Au/Toy@G3 NGs + UTMD + Anti‐PD‐L1 group (0.36%), demonstrating an effective reversal of the immunosuppressive TME after the combined treatment. In addition, immunofluorescence staining was performed to evaluate the distribution of active T cells in spleens (Figure 6g and Figure S23, Supporting Information). The distribution of CD4^+^ and CD8^+^ T cells in Au/Toy@G3 NGs + UTMD + Anti‐PD‐L1 group is the highest among all groups (p < 0.05) , suggesting the best immune response realized by augmented chemoimmunotherapy.

In addition, the treatment of Au/Toy@G3 NGs + UTMD + Anti‐PD‐L1 results in the highest expression level of antitumor‐related cytokines such as TNF‐*α* and interferon‐*γ* (IFN‐*γ*) among all groups (*p* < 0.001), confirming the best antitumor immune response of the combination therapy (Figure S24, Supporting Information). These results indicate that the constructed multifunctional Au/Toy@G3 NGs combined with UTMD and Anti‐PD‐L1 treatment can effectively remodulate immunosuppressive TME to enhance the immunotherapeutic effect of tumors by increasing the proportion of CTLs and M1‐type TAMs as well as reducing the proportion of Tregs and M2‐type TAMs.

### 2.11 Biosafety Evaluations

To evaluate the biosafety of Au/Toy@G3 NGs + UTMD + Anti‐PD‐L1, major organs (heart, liver, spleen, lung, and kidney) of tumor‐bearing mice were collected after14 days of various treatments and observed by H&E staining (Figure S25, Supporting Information). No significant damages can be observed for all major organs after different treatments, similar to the PBS group. Combined with the body weight changes recorded in Figure 5b, it can be concluded that all materials utilized in this study possess good biocompatibility and show inconspicuous side effects to mice.

Furthermore, healthy mice were intravenously injected with PBS, free Toy, or Au/Toy@G3 NGs, and blood samples were collected after one week for blood routine and blood biochemical analysis (Figure S26, Supporting Information). The platelet (PLT) level and uric acid (UA) level in the free Toy group is out of the normal range, suggesting that the treatment of free Toy may cause some coagulation abnormalities and abnormal renal function in mice. In contrast, there are no appreciable changes in red blood cells (RBC), platelets (PLT), white blood cells (WBC), lymphocytes (Lymph), alanine aminotransferase (ALT), aspartate aminotransferase (AST), creatinine (CR) and uric acid (UA) in the Au/Toy@G3 NGs group, which is similar to the PBS control group. These results further indicate that the Au/Toy@G3 NGs have good biological safety.

## 3 Conclusion

We here developed an advanced intelligent nanomedicine formulation based on redox‐responsive dendrimer NGs that can be co‐loaded with Au NPs and Toy for CT imaging‐guided and UTMD‐facilitated combination chemoimmunotherapy of tumors. The created G3‐PEG‐SAT dendrimers can act as a macromonomer to generate redox‐responsive G3 NGs through disulfide bonded self‐crosslinking and a reverse microemulsion method for in situ loading of Au NPs and physical encapsulation of Toy. The prepared Au/Toy@G3 NGs having an average size of 193 nm exhibit good colloidal stability, excellent biocompatibility, and redox responsiveness to TME with relatively high GSH concentration that can trigger the synchronous fast release of Au NPs and Toy to exert antitumor effects in vitro and in vivo through Au NP‐mediated TAM polarization and Toy‐induced chemotherapy through ERS amplification. Meanwhile, the growth inhibition of cancer cells in vitro and tumors in vivo treated with the Au/Toy@G3 NGs can be significantly enhanced under the assistance of UTMD technology due to the sonoporation‐induced improved cell uptake and tumor penetration. Through a multi‐pronged strategy to modulate immune cells including the maturation of DCs through ICD by Toy‐mediated ERS amplification for subsequent activation of tumor‐infiltrating CD4^+^ and CD8^+^ T cells and reduction of Tregs in tumors, Au NPs‐mediated M2‐type TAM repolarization, and Anti‐PD‐L1‐mediated ICB therapy for further activation of T cells, enhanced chemoimmunotherapy of pancreatic tumor model can be realized under the assistance of UTMD. The designed intelligent Au/Toy@G3 NGs combined with the UTMD and ICB may be regarded as a promising nanomedicine formulation for CT imaging and enhanced chemoimmunotherapy of tumors, which is able to address some current limitations of cancer theranostics for clinical oncology applications.

## 4 Experimental Section

### Synthesis of G3‐PEG‐SAT

First, NHS‐PEG‐SAT (35 mg) was dissolved in DMSO (300 µL), then added dropwise to a solution of G3.NH_2_ (12.1 mg, in 200 µL methanol) at a G3.NH_2_/PEG molar ratio of 1: 10, followed by the addition of catalyst DIEA (5 µL) while stirring at room temperature for 2 h. Subsequently, ether was added to the above mixture until it turned milky white and the organic solvent was removed by centrifugation at 20379 g for 10 min. The precipitate obtained after centrifugation was washed twice with methanol/ether (1: 5, v/v) to remove any unreacted substances to obtain the G3‐PEG‐SAT product.

### Synthesis of Au/Toy@G3 NGs

The prepared G3‐PEG‐SAT was dissolved in 1 mL phosphate buffered saline (PBS, as a water phase), added dropwise to a solution of Span80 and Tween80 (5: 1 of mass ratio, 280 mg) in 12 mL n‐hexane (as an oil phase) in an ice bath, and mixed by sonication (XL2000 Misonix Sonicator, Newtown, CT) for 1 min at a power of 20 W to form an emulsion. Then, NH_2_OH·HCl (1 mg, in 100 µL PBS) was added to the above emulsion under stirring for 8 h at room temperature. After that, the solution was dialyzed against water (9 times, 2 L) using a dialysis membrane with an MWCO of 3000 Da for 3 days to obtain the G3 PAMAM dendrimer‐based NGs (G3 NGs).

Next, Au nanoparticles (NPs) were loaded into G3 NGs in situ through a rapid sodium borohydride reduction method. Specifically, HAuCl_4_·4H_2_O solution (30 mg mL^−1^, 34 µL in water) was added dropwise to an aqueous solution of G3 NGs (2 mg) at an Au/G3.NH_2_ feeding molar ratio of 10:1 under stirring for 30 min under an ice bath, followed by the rapid addition of NaBH_4_ (1 mg, in 100 µL water). The mixture solution was stirred for 3 h, dialyzed against water (9 times, 2 L) through a dialysis membrane with an MWCO of 3000 Da for 3 days to obtain the product of Au NP‐loaded G3 NGs (for short, Au@G3 NGs).

Then, Toy (ranging from 0.4‐2 mg) was dissolved in water, and mixed with the solution of Au@G3 NGs (2 mg mL^−1^, 2 mL in water) at different G3.NH_2_/Toy mass ratios (1:0.1, 1:0.25, or 1:0.5). Each mixture was stirred for 8 h at room temperature. After that, the solution was dialyzed against water using a dialysis membrane with an MWCO of 3000 Da for 3 days to obtain the Toy‐loaded Au@G3 NGs (for short, Au/Toy@G3 NGs).

### Cell Culture and In Vitro Assays

Pan02 cells (a murine pancreatic cancer cell line), RAW 264.7 cells (a mouse macrophage cell line), and DCs were regularly cultured, passaged, and adopted for in vitro assays including cytotoxicity, cellular uptake, cell apoptosis, ERS, RT‐PCR, WB, macrophage polarization, and ICD induction.

### Animal Experiments

All animal experiments were performed following the protocols approved by the Ethical Committee for Experimental Animal Care and Use of Donghua University (approval number: DHUEC‐STCSM‐2020‐07) and also in accordance with the policy of the National Ministry of Health of China. The Mouse Pan02 tumor model was established for investigations of CT imaging, pharmacokinetics, biodistribution, and in vivo UTMD‐facilitated chemoimmunotherapy. Histological examinations of major organs, hematology, and serum biochemistry analysis were used to evaluate the biosafety of the Au/Toy@G3 NGs.

### Statistical Analysis

All experimental data were represented as the mean ± standard deviation through at least three experiments. One‐way analysis of variance statistical method was used to analyze the experimental results through IBM SPSS Statistic 25 software (IBM, Armonk, NY). A p value of 0.05 was selected as a significance level, and the data were indicated with (*) for *p* < 0.05, (**) for *p* < 0.01, and (***) for *p* < 0.001, respectively. Full experimental details can be seen in the Supporting Information.

## Conflict of Interest

The authors declare no conflict of interest.

## Supporting information

Supporting Information

## Data Availability

Research data are not shared.
