# Supplementary material for: Redox‐Responsive Dendrimer Nanogels Enable Ultrasound‐Enhanced Chemoimmunotherapy of Pancreatic Cancer via Endoplasmic Reticulum Stress Amplification and Macrophage Polarization
Source: Adv Sci (Weinh). 2023 Jun 23;10(24):2301759. doi: 10.1002/advs.202301759 (PMC10460845; doi:10.1002/advs.202301759)
Supplement: Supplementary file 1 — Supporting Information [file ADVS-10-2301759-s001.pdf]

ADVANCED SCIENCE
Open Access

Supporting Information

for *Adv. Sci.*, DOI 10.1002/advs.202301759

Redox-Responsive Dendrimer Nanogels Enable Ultrasound-Enhanced Chemoimmunotherapy of Pancreatic Cancer via Endoplasmic Reticulum Stress Amplification and Macrophage Polarization

Guizhi Zhang, Mengsi Zhan, Changchang Zhang, Zhiqiang Wang, Huxiao Sun, Yuchen Tao, Qiusheng Shi, Meijuan He, Han Wang, João Rodrigues, Mingwu Shen* and Xiangyang Shi**

Supporting Information

Redox-Responsive Dendrimer Nanogels Enable Ultrasound-Enhanced Chemoimmunotherapy of Pancreatic Cancer *via* Endoplasmic Reticulum Stress Amplification and Macrophage Polarization

Guizhi Zhang, Mengsi Zhan, Changchang Zhang, Zhiqiang Wang, Huxiao Sun, Yuchen Tao, Qiusheng Shi, Meijuan He, Han Wang, João Rodrigues, Mingwu Shen*, Xiangyang Shi**

G. Z. Zhang, M. S. Zhan, C. C. Zhang, Z. Q. Wang, H. X. Sun, Prof. M. W. Shen, Prof. X. Y. Shi
State Key Laboratory for Modification of Chemical Fibers and Polymer Materials, Shanghai Engineering Research Center of Nano-Biomaterials and Regenerative Medicine, College of Biological Science and Medical Engineering, Donghua University, Shanghai 201620, China
E-mail: mwshen@dhu.edu.cn (M. Shen) and xshi@dhu.edu.cn (X. Shi)

Y. C. Tao, Prof. Q. S. Shi
Department of Ultrasound, Shanghai General Hospital, Shanghai Jiao Tong University School of Medicine, Shanghai 200080, China

M. J. He, Prof. H. Wang
Department of Radiology, Shanghai General Hospital, Shanghai Jiao Tong University School of Medicine, Shanghai 200080, China

Prof. J. Rodrigues, Prof. X. Y. Shi
CQM - Centro de Química da Madeira, MMRG, Universidade da Madeira, Campus Universitário da Penteada, 9020-105 Funchal, Portugal
E-mail: joaor@uma.pt/joaoc@staff.uma.pt

Keywords: dendrimer nanogels; redox-responsiveness; immunogenic cell death; ultrasound-targeted microbubble destruction; chemoimmunotherapy

Experimental Section

Materials: Generation 3 (G3) poly(amidoamine) (PAMAM) dendrimers ($G3.NH_2$) were purchased from Dendritech (Midland, MI). N, N-Diisopropylethylamine (DIEA) was from Aladdin Biochemical Technology Co., Ltd. (Shanghai, China). Sorbitan monooleate (Span 80) was from Sigma Aldrich (St. Louis, MO). NHS-PEG-SAT (Mw = 2000) was from Hunan Huateng Pharmaceutical Co., Ltd. (Changsha, China). $NH_2OH\cdot HCl$ and sodium borohydride ($NaBH_4$) were from J&K Scientific Ltd. (Shanghai, China). Toyocamycin (Toy) was from Shanghai Wei Huan Biological Technology Co., Ltd. (Shanghai, China). SonoVue microbubbles (MBs) were from Bracco Diagnostics Inc. (Geneva, Switzerland). Gold (III) chloride tetrahydrate ($HAuCl_4\cdot 4H_2O$) and all the other chemicals were acquired from Sinopharm Chemical Reagent Co., Ltd. (Shanghai, China) or other commercial resources. Regenerated cellulose dialysis membranes with a molecular weight cut-off (MWCO) of 3000 Da were acquired from Shanghai Yuanye Biotechnology Co., Ltd. (Shanghai, China). Pan02 cells (a murine pancreatic cancer cell line) and RAW 264.7 cells (a mouse macrophage cell line) were provided by the Institute of Biochemistry and Cell Biology, the Chinese Academy of Sciences (Shanghai, China). Dendritic cells (DCs) were from Shanghai Cancer Center, Fudan University. Sodium dodecyl sulfate (SDS) sample loading buffer and SDS-polyacrylamide gel were from Tanon Science & Technology Co., Ltd. (Shanghai, China). Cell counting kit-8 (CCK-8) was from 7sea Biotech. Co., Ltd. (Shanghai, China). 4',6-Diamidino-2-phenylindole (DAPI) was acquired from BestBio Biotechnology Co., Ltd. (Shanghai, China). Dulbecco's Modified Eagle Medium (DMEM), fetal bovine serum (FBS), penicillin, and streptomycin were from Gino Biomedical Technology Co., Ltd. (Hangzhou, China). Annexin V-fluorescein isothiocyanate (FITC)/propidium iodide (PI) apoptosis detection kit was from KeyGEN BioTECH. (Beijing, China). BeyoR^{TM} first strand cDNA synthesis kit, total RNA extractor (Trizol), BeyoFast^{TM} SYBR Green qPCR Mix (2×, Low ROX), anti-

glucose-regulated protein 78 (GRP78) rabbit polyclonal antibody (pAb), anti-pIRE1α rabbit pAb, anti-unsplicing X-box binding protein 1 (XBP1u) rabbit pAb, anti-splicing X-box binding protein 1 (XBP1s) rabbit pAb, anti-C/eBP homologous protein (CHOP) monoclonal antibody (mAb), and anti-calreticulin (CRT) rabbit pAb were from Beyotime Biotechnology Co., Ltd. (Shanghai, China). Mouse high mobility group protein 1 (HMGB-1) enzyme-linked immunosorbent assay (ELISA) kit was from ZCIBIO Technology Co., Ltd. (Shanghai, China) and adenosine triphosphate (ATP) ELISA kit was acquired from Beyotime Biotechnology Co., Ltd. (Shanghai, China). Programmed cell death ligand 1 (PD-L1) antibody (Anti-PD-L1) was from Bio X Cell (West Lebanon, NH). The anti-CD80-PE mAb, anti-CD86-FITC mAb, anti-CD86-PE mAb, anti-CD206-FITC mAb, anti-CD4-FITC mAb, anti-CD8 alpha-PE mAb, anti-CD4-PE mAb, anti-CD25-FITC mAb, anti-Foxp3-APC mAb, and anti-MHC class I (MHC-I)-PE mAb were from Thermo Fisher Scientific (Waltham, MA). Tumor-infiltrating lymphocyte cell separation medium kit for mice was from Beijing Solarbio Science & Technology Co., Ltd. (Beijing, China). Water used in all experiments was purified using a PURIST UV Ultrapure Water system (RephiLe Bioscience, Ltd., Shanghai, China) with a resistivity higher than 18.2 MΩ·cm.

Synthesis of G3-PEG-SAT: Firstly, NHS-PEG-SAT (35 mg) was dissolved in DMSO (300 μL), then added dropwise to a solution of G3.NH_2 (12.1 mg, in 200 μL methanol) at a G3.NH_2/PEG molar ratio of 1: 10, followed by the addition of catalyst DIEA (5 μL) while stirring at room temperature for 2 h. Subsequently, ether was added to the above mixture until it turned milky white and the organic solvent was removed by centrifugation at 20379 g for 10 min. The precipitate obtained after centrifugation was washed twice with methanol/ether (1: 5, v/v) to remove any unreacted substances to obtain the G3-PEG-SAT product.

Synthesis of Au/Toy@G3 NGs: The prepared G3-PEG-SAT was dissolved in 1 mL phosphate buffered saline (PBS, as a water phase), added dropwise to a solution of Span80 and Tween80 (5: 1 of mass ratio, 280 mg) in 12 mL n-hexane (as an oil phase) in an ice bath,

and mixed by sonication (XL2000 Misonix Sonicator, Newtown, CT) for 1 min at a power of 20 W to form an emulsion. Then, $NH_2OH \cdot HCl$ (1 mg, in 100 μL PBS) was added to the above emulsion under stirring for 8 h at room temperature. After that, the solution was dialyzed against water (9 times, 2 L) using a dialysis membrane with an MWCO of 3000 Da for 3 days to obtain the G3 PAMAM dendrimer-based NGs (G3 NGs).

Next, Au nanoparticles (NPs) were loaded into G3 NGs *in situ* through a rapid sodium borohydride reduction method. Specifically, $HAuCl_4 \cdot 4H_2O$ solution (30 mg/mL, 34 μL in water) was added dropwise to an aqueous solution of G3 NGs (2 mg) at an Au/G3.NH_2 feeding molar ratio of 10: 1 under stirring for 30 min under an ice bath, followed by the rapid addition of $NaBH_4$ (1 mg, in 100 μL water). The mixture solution was stirred for 3 h, and dialyzed against water (9 times, 2 L) through a dialysis membrane with an MWCO of 3000 Da for 3 days to obtain the product of Au NP-loaded G3 NGs (for short, Au@G3 NGs).

Then, Toy (ranging from 0.4-2 mg) was dissolved in water, and mixed with the solution of Au@G3 NGs (2 mg/mL, 2 mL in water) at different G3.NH_2/Toy mass ratios (1: 0.1, 1: 0.25, or 1: 0.5). Each mixture was stirred for 8 h at room temperature. After that, the solution was dialyzed against water using a dialysis membrane with an MWCO of 3000 Da for 3 days to obtain the Toy-loaded Au@G3 NGs (for short, Au/Toy@G3 NGs). The drug encapsulation efficiency (EE) and drug loading content (LC) were calculated according to the following equations:

$$EE\ (\%) = (M_t/M_0) \times 100\% \qquad \text{(S1)}$$

$$LC\ (\%) = (M_t/M_L) \times 100\% \qquad \text{(S2)}$$

where M_t, M_0, and M_L stand for the masses of loaded Toy within the NGs, the initial mass of Toy, and the mass of Toy-loaded NGs, respectively.

Characterization Techniques: 1H NMR was performed using a Bruker NMR (400 MHz) spectrometer. The G3-PEG-SAT was dissolved in D_2O (1 mg/mL) before measurements. The morphology of the Au/Toy@G3 NGs was observed by transmission

electron microscopy (TEM) using a JEOL2010F analytical electron microscope (JEOL, Tokyo, Japan) at an operating voltage of 200 kV. The sample was prepared by dropping an NG water suspension (0.2 mg/mL, 5 μL) onto a carbon-coated copper grid, and air-dried before measurements. Hydrodynamic size and zeta potential measurements were performed using a Malvern Zetasizer (Nano ZS model ZEN3600, Worcestershire, UK) coupled with a standard 633-nm laser. G3 NGs, Au@G3 NGs, or Au/Toy@G3 NGs were dispersed in water at a concentration of 0.2 mg/mL before measurements. To examine the stability of the prepared Au@G3 NGs and Au/Toy@G3 NGs dispersed in H_2O, PBS, or cell culture medium (DMEM with 10% FBS), dynamic light scattering was performed to measure their hydrodynamic size changes for seven consecutive days. To investigate the stability of Toy in the Au/Toy@G3 NGs, the Au/Toy@G3 NGs were dispersed in H_2O, PBS or cell culture medium (DMEM with 10% FBS) to test the concentration of Toy in the supernatant after centrifugation for seven consecutive days through UV-vis spectroscopy. UV-vis spectra were acquired using a Lambda 25 UV-vis spectrophotometer (Perkin Elmer, Waltham, MA). All samples (0.2 mg/mL) were dispersed in water before measurements. The content of Au was determined by Leeman Prodigy inductively coupled plasma-optical emission spectroscopy (ICP-OES, Hudson, NH). The prepared Au@G3 NGs (100 μL, 1 mg/mL) were digested with aqua regia (1.9 mL), which was made by mixing nitric acid and hydrochloric acid at a volume ratio of 1: 3. Later, the solution was diluted with 3 mL of water to determine the Au content in the Au@G3 NGs.

***In Vitro* Toy Release from the Au/Toy@G3 NGs**: Redox-responsive Toy release from the Au/Toy@G3 NGs was evaluated under different conditions. The Au/Toy@G3 NGs ([G3] = 1.23 mg/mL, [Toy] =182 μg/mL) were dispersed in phosphate buffer at different pHs (pH 7.4 or 6.5) in the presence or absence of GSH (10 mM), then transferred into a dialysis bag (MWCO = 3000 Da), and submerged into 9 mL of the corresponding buffer medium to have

final NG and Toy concentrations at 0.123 mg/mL and 18.2 μg/mL, respectively. The whole system was kept under a constant temperature vibration shaker at 37 °C for 0.25, 0.5, 1, 2, 3, 4, 6, 8, 12, 24, 36, 48, 60, and 72 h, respectively. Then, 1 mL of the buffer medium was pipetted out at each scheduled time interval and the corresponding outer phase was maintained constant by replenishing the same volume of the corresponding buffer medium. The Toy concentration was determined by UV-vis spectrometry to quantify the Toy absorption at 280 nm. The experiment was performed in triplicate for each sample.

***In Vitro* Ultrasound-Targeted Microbubble Destruction (UTMD) Technology:** The UTMD treatment of Pan02 cells was conducted using Chattanooga Intelect Mobile Ultrasound Reference 2776 with a 5 cm^2 applicator (DJO France SAS, Mouguerre, France). After Pan02 cells reached 80~90% confluence, they were digested, collected, and counted. According to our previous work,[1] the operation procedure was set as follows: Pan02 cells were co-cultured with each formulation and SonoVue microbubbles (59 mg of SF_6 gas, 25 mg of microbubble powder, and 5 mL of sterile normal saline (NS) solution were mixed and shaken for about 20 s to form a white microbubble suspension), and the bottom of the cell culture plate was coated with an aqueous coupling agent, subsequently exposed to the ultrasound apparatus under the following optimized parameters: 0.4 W/cm^2, 1 MHz, 20% MBs, PRF 1 kHz, and 30 s according to the literature.[2] The working concentration of SonoVue MBs was 20% (v/v). The SonoVue MB suspension was prepared 15 min in advance, placed at 4 ^oC, and shaken to form homogeneous white emulsion before use. To investigate the UTMD-promoted cytotoxicity of the NGs, the seeded cells were treated with medium containing NGs and 20% SonoVue, UTMD treated for 30 s, and further incubated for 24 h before regular cell viability assay (see below). For *in vitro* cellular uptake experiments, the processed cell suspension was seeded into 12-well plates for 6 h to study the influence of UTMD on the cellular uptake behavior of NGs under the same UTMD conditions described above.

Cell Culture and Cytotoxicity Assay: Pan02 cells were regularly cultured and passaged in DMEM supplemented with 10% FBS and 1% penicillin-streptomycin in a Thermo Scientific cell incubator (Waltham, MA) at 37 °C and 5% CO_2. CCK-8 assay was carried out to analyze the cytotoxicity of Au@G3 NGs, Toy, Toy + UTMD, Au/Toy@G3 NGs, and Au/Toy@G3 NGs + UTMD. Firstly, Pan02 cells were seeded in a 96-well plate at a density of 1×10^4 cells per well with 0.1 mL complete DMEM for each well and cultured for 24 h. After that, the medium was replaced with fresh complete medium containing different NGs at various Toy or corresponding Toy concentrations (0.1, 0.25, 0.5, 0.75, 1, 2.5, 5, and 10 μg/mL, respectively), and the cells were incubated for 24 h. Then, the medium in each well was taken out, and the cells were washed with PBS three times and incubated with 0.1 mL serum-free medium containing 10% CCK-8 for an additional 2 h. Finally, the absorbance of each well was measured by a Thermo Scientific Multiskan MK3 ELISA reader (Waltham, MA) at 450 nm. The half maximal inhibitory concentration (IC_{50s}) of free Toy, Toy + UTMD, Au/Toy@G3 NGs, and Au/Toy@G3 NGs + UTMD were calculated using a GraphPad Prism software (GraphPad Software Inc., San Diego, CA). At the same time, to investigate the UTMD-enhanced cytotoxicity of Toy and Au/Toy@G3 NGs, the Pan02 cells were suspended in medium mixed with 20% SonoVue MBs solution and 80% fresh DMEM containing Toy or Au/Toy@G3 NGs at different Toy concentrations (the same as above). Meanwhile, the Pan02 cells in the 96-well plate were subjected to UTMD treatment (0.4 W/cm^2, 30 s per well). The cytotoxicity was then measured following the same procedures mentioned above. For each sample, 6 parallel wells were tested to give a mean value and standard deviation.

Cellular Uptake Assay: The cellular uptake behavior of Au/Toy@G3 NGs in the presence or absence of UTMD treatment was explored by ICP-OES. Briefly, Pan02 cells were seeded into a 12-well plate at a density of 1×10^5 cells/well with 1.0 mL medium and incubated overnight. The next day, the medium in each well was substituted with fresh medium containing Au/Toy@G3 NGs with different Au concentrations (0.9, 1.8, 3.6, or 9 μM,

and the corresponding Toy concentrations were 1.0, 2.1, 4.2, or 10.4 μg/mL), and the cells were then incubated for 6 h. The culture medium was removed and the cells were washed with PBS for 3 times. The adherent Pan02 cells were digested, centrifuged, and redispersed in PBS (1 mL). The collected cells were digested by *aqua regia* solution for 4 h, diluted with water, and analyzed by ICP-OES to quantify the Au content. For the Au/Toy@G3 NGs + UTMD group, Pan02 cells were seeded in 12-well plates (1×10^5 cells per well) with 1 mL fresh medium containing Au/Toy@G3 NGs with different final Au concentrations (0.9, 1.8, 3.6, and 9 μM, respectively and the corresponding Toy concentrations were 1.0, 2.1, 4.2, and 10.4 μg/mL, respectively) and 20% of Sonovue MBs suspension and immediately subjected to UTMD treatment (0.4 W/cm^2, 30 s per well), followed by 6 h incubation. The measurement procedure to verify UTMD-enhanced cellular uptake was the same as that described above. For each sample, 3 parallel wells were tested to give a mean value and standard deviation.

Cell Apoptosis Assay: The apoptosis of Pan02 cells treated by different NGs was examined using an Annexin V-FITC/PI apoptosis detection kit. In brief, Pan02 cells were cultured in 6-well plates, incubated with PBS, Au@G3 NGs, Toy, Toy + UTMD, Au/Toy@G3 NGs or Au/Toy@G3 NGs + UTMD ([Toy] = 5 μg/mL for all Toy-related groups, and the concentration of Au@G3 NGs corresponded to the Toy-incorporated groups) for 12 h. Subsequently, the cells in each well were washed, collected, resuspended in 195 μL of binding buffer, and added with 5 μL of Annexin V-FITC and 5 μL of PI according to the kit instruction. Then, the samples were incubated for 15 min at room temperature in the dark before flow cytometry analysis. Cells treated with PBS were used as a control. For each sample, 1×10^4 cells were counted and each measurement was repeated for 3 times.

Endoplasmic Reticulum Stress (ERS) Assay: Quantitative real-time polymerase chain reaction (RT-PCR) was performed to analyze the expression levels of ERS-related genes, including glucose-regulated protein 78 (*GRP78*), unsplicing X-box binding protein 1 (*XBP1u*), splicing X-box binding protein 1 (*XBP1s*) and C/eBP homologous protein (*CHOP*).

In brief, Pan02 cells were seeded and treated with PBS, Au@G3 NGs, Toy, Toy + UTMD, Au/Toy@G3 NGs or Au/Toy@G3 NGs + UTMD ([Toy] = 5 μg/mL for all Toy-related groups, and the concentration of Au@G3 NGs corresponded to the Toy-containing groups) for 6 h. After that, the cells were washed with PBS for three times and collected for RNA extraction using an RNAeasy Plus Animal RNA Isolation Kit. Then, the first strand cDNA was synthesized by BeyoRT™ III First Strand cDNA Synthesis Kit according to the manufacturer's instructions. Subsequently, the obtained cDNA was mixed with a specific primer (listed in Table S4) and BeyoFast™ SYBR-Green qPCR Mix to analyze the expression of *GRP78*, *XBP1u*, *XBP1s*, and *CHOP* by RT-PCR (7500, Applied Biosystems, Waltham, MA), respectively. The amplification was performed for 40 cycles by a fluorescence detection system with SYBR green fluorescence. Each cycle consisted of heat denaturation at 95 °C for 15 s, annealed at 62 °C for 15 s, and extended at 72 °C for 30 s, and each sample was quantified by a comparative cycle threshold method to calculate the relative gene expression using the reference gene *β-actin*. Cells treated with PBS were used as a control. For each sample, the measurement was repeated 3 times.

Western blot (WB) assay was also performed to analyze the expression of ERS-related proteins (GRP78, XBP1u, XBP1s, pIRE1α, and CHOP). Briefly, Pan02 cells were regularly cultured in 6-well plates and incubated with PBS, Au@G3 NGs, Toy, Toy + UTMD, Au/Toy@G3 NGs or Au/Toy@G3 NGs + UTMD ([Toy] = 1.7 μg/mL for all Toy-related groups, and the concentration of Au@G3 NGs corresponded to the Toy-containing groups) for 24 h. Thereafter, cells in each group were collected, washed, and incubated with 0.2 mL cell lysis buffer (containing 1% PMSF) in an ice bath for 30 min. The lysates were analyzed through WB to detect the expression levels of GRP78, XBP1u, XBP1s, pIRE1α, and CHOP according to the literature.[3] β-actin was employed as a reference protein.

Evaluation of Macrophage Repolarization Effect *in Vitro*: RAW 264.7 cells were used as a model to evaluate the effect of different NGs on macrophage repolarization. To be

brief, RAW 264.7 cells were seeded into 6-well plates at a density of 2 × 10^5 cells per well and incubated overnight. Then, the cells were washed three times with PBS, and the medium was replaced with fresh medium containing 50 ng/mL interleukin-4 (IL-4) except for the PBS group and cultured for an additional 24 h to polarize RAW 264.7 cells to M2-type as a negative control. After that, the culture medium in each well was replaced by fresh DMEM containing PBS, Au@G3 NGs, Toy, Toy + UTMD, Au/Toy@G3 NGs, Au/Toy@G3 NGs + UTMD ([Toy] = 5 μg/mL for all Toy-related groups, and the concentration of Au@G3 NGs corresponded to the Toy-incorporated groups), or lipopolysaccharide (LPS, 2 μg/mL) and cells were cultured for additional 24 h. LPS was used as a positive control. The cells were washed, digested, centrifuged, and resuspended in PBS, followed by staining with FITC-CD206 and PE-CD86 antibodies in the dark for 15-20 min in an ice bath. Thereafter, the cell suspension was centrifuged, washed three times with PBS, and resuspended in 500 μL of PBS before flow cytometry analysis. The fluorescence intensity of the M2-type marker anti-FITC-CD206 and the M1-type marker anti-PE-CD86 antibodies were measured, respectively.

***In Vitro* Assays of Immunogenic Cell Death (ICD):** To verify the ICD effect of Pan02 cells induced by different treatments, the expression level of CRT on the cell surface was checked through immunofluorescence staining. To be brief, Pan02 cells were seeded in confocal dishes at a density of 2 × 10^5 cells per dish with 1.0 mL medium and incubated overnight. Then, the culture medium in each dish was replaced by fresh DMEM containing PBS, Au@G3 NGs, Toy, Toy + UTMD, Au/Toy@G3 NGs or Au/Toy@G3 NGs + UTMD ([Toy] = 5 μg/mL for all Toy-related groups, and the concentration of Au@G3 NGs corresponded to the Toy-incorporated groups) and cells were cultured for 24 h. The cells treated with PBS were used as control. Then, the cells were washed with PBS for three times and fixed with glutaraldehyde (2.5%) for 15 min. After that, the cells were treated with immunostaining blocking buffer for 60 min, incubated with anti-CRT (primary antibody) for another 60 min, washed with PBS, and incubated with FITC-labeled secondary antibody for

60 min. Finally, the cells were stained with DAPI for 15 min at 37 °C and imaged by confocal microscopy to observe CRT expression according to protocols reported in the literature.[4]

Then, the secretion of ATP and the release of HMGB-1 were detected. In brief, Pan02 cells were seeded in 6-well plates at a density of 2 × 10^5 cells per well with 1 mL medium. After 24 h incubation, the culture medium in each well was replaced by fresh DMEM containing Au@G3 NGs, Toy, Toy + UTMD, Au/Toy@G3 NGs or Au/Toy@G3 NGs + UTMD ([Toy] = 5 μg/mL for all Toy-related groups, and the concentration of Au@G3 NGs corresponded to the Toy-incorporated groups) and cells were cultured for additional 24 h. The cells treated with PBS were used as control. After that, the culture medium from each well was collected and used for the analysis of ATP using an ATP detection kit (Beyotime Biotechnology Co., Ltd., Shanghai, China) and the detection of HMGB-1 using an HMGB-1 ELISA assay kit according to the manufacturer's instruction, respectively.

In Vitro **ICD-Induced Maturation of Dendritic Cells (DCs):** To investigate the ICD-induced maturation of DCs, a 6-well transwell system with 0.4-μm polycarbonate porous membranes was used. Briefly, Pan02 cells were seeded in the upper wells at a density of 2 × 10^5 cells per well with 1 mL medium and incubated overnight. Then, the culture medium in each upper well was replaced by fresh DMEM containing PBS, Au@G3 NGs, Toy, Toy + UTMD, Au/Toy@G3 NGs or Au/Toy@G3 NGs + UTMD ([Toy] = 5 μg/mL for all Toy-related groups, and the concentration of Au@G3 NGs corresponded to the Toy-incorporated groups) and cells were cultured for additional 24 h. Meanwhile, DCs were seeded in the lower wells at a density of 2 × 10^5 cells per well with 1 mL medium and incubated for 24 h. Subsequently, the Pan02 cells in the upper wells were merged with the lower wells for the mixed culture of both Pan02 cells and DCs. After 24 h incubation, DCs were digested, collected, and stained with anti-CD86-FITC and anti-CD80-PE antibodies for 20 min in the dark. Finally, the DCs were washed with PBS and resuspended in 300 μL of PBS for flow

cytometry assay. For each sample, 1 × 10^4 cells were counted and each measurement was repeated for 3 times.

To investigate the expression of MHC-I of DCs induced by ICD *in vitro*, the DCs were treated according to the same experimental procedures as above, and then digested, collected, and stained with MHC-I-PE antibody for 15 min. Then, the DCs were washed with PBS and resuspended in 300 μL of PBS before flow cytometry assays. For each sample, 1 × 10^4 cells were counted and each measurement was repeated for 3 times.

PD-L1 Expression of Cancer Cells *in Vitro*: To investigate the expression of PD-L1 on Pan02 cells after different treatments, the Pan02 cells were regularly cultured in 6-well plates and treated with PBS, Au@G3 NGs, Toy, Toy + UTMD, Au/Toy@G3 NGs or Au/Toy@G3 NGs + UTMD ([Toy] = 1.7 μg/mL for all Toy-related groups, and the concentration of Au@G3 NGs corresponded to the Toy-containing groups) for 24 h. Then, the cells were collected and analyzed by WB according to protocols used in ERS assay to evaluate the PD-L1 expression. Cells treated with PBS were used as control. GAPDH was employed as a reference protein.

Hemolysis Assay: All animal experiments were approved by the Ethical Committee of Experimental Animal Care and Use of Donghua University (approval number: DHUEC-STCSM-2020-07), and performed following the protocols of the same committee and also in accordance with the policy of the National Ministry of Health of China. Hemolysis assay was performed to evaluate the hemocompatibility of the Au/Toy@G3 NGs according to the literature.[5] In brief, mouse blood collected from the inner canthus vein plexus was stabilized with heparin and centrifuged (936 g, 5 min). Then the precipitate was washed with normal saline (NS) 3 times to remove the serum and collect the pure red blood cells (RBCs) *via* repeated centrifugation/redispersion processes (936 g, 5 min). The obtained RBCs were then 50 times diluted with NS. After that, 500 μL of the RBC suspension was mixed with 500 μL NS (negative control), water (positive control), or NS solution of Au/Toy@G3 NGs at

different Toy concentrations (0.25, 0.5, 1, 2.5, and 5 μg/mL, respectively). After 2 h incubation at 37 °C, all samples were centrifuged at 10397 g for 5 min. The photos of the samples were taken, and the absorbance of the obtained supernatants was measured by UV-vis spectrometry at 540 nm. The hemolysis rate was calculated according to the following equation:

$$\text{Hemolysis rate}(\%) = \frac{A_S - A_B - A_N}{A_P - A_N} \times 100\% \qquad \text{(S3)}$$

where A_S, A_B, A_N and A_P represent the absorbance of the samples, the initial Au/Toy@G3 NGs with corresponding concentration, the negative control and the positive control.

Pharmacokinetics: To explore the pharmacokinetics of the Au/Toy@G3 NGs *in vivo*, healthy C57BL/6 mice were intravenously (i.v.) administrated with them ([Au] = 4 mM, in 100 μL of PBS for each mouse). The blood samples at various time points (0, 1, 2, 4, 8, 12, 24, 48, and 72 h, respectively) were collected, weighed, and digested by *aqua regia* solution for 24 h. Each sample was diluted with water, and the Au content was quantified by ICP-OES. The blood circulation half-decay time was determined using a one-phase decay exponential model by GraphPad prism® 8.0 software.

X-Ray Attenuation Property: The X-ray attenuation property of the Au/Toy@G3 NGs was studied using a computed tomography (CT) system (Brilliance iCT, Haifa, Israel) at 70 kV and a slice thickness of 1 mm. The X-ray attenuation intensity of Au/Toy@G3 NGs with different Au concentrations (5, 10, 20, 30, and 60 mM, respectively) was determined. For each sample, the X-ray attenuation intensity was determined in Hounsfield units (HU) by a standard display program.

***In Vivo* CT Imaging and Biodistribution:** Female C57BL/6 mice with 5-6 weeks old (body weight of 15-20 g for each mouse) were obtained from the Shanghai Slac Laboratory Animal Center (Shanghai, China). The xenograft tumor model was established by injection of 2×10^6 cells Pan02 cells (in 100 μL of PBS) into the right hind leg of each C57BL/6 mouse.

When the tumor volume reached about 100 mm^3, Pan02 tumor-bearing mice were divided into two groups of Au/Toy@G3 NGs or Au/Toy@G3 NGs + UTMD (1 MHz, 0.4 W/cm^2, 20% MBs, 2 min) for CT imaging (n = 3 for each group). Each tumor-bearing mouse was intravenously injected with Au/Toy@G3 NGs or Au/Toy@G3 NGs + UTMD ([Au] = 10 mM, in 0.1 mL PBS) *via* the tail vein. Then, the mice were scanned at 0, 15, 30, 60, 90, 120, 150, or 180 min post-injection through a CT system (Brilliance iCT, Haifa, Israel) at 70 kV and a slice thickness of 1 mm. After CT imaging, the mice were sacrificed, and the major organs (heart, liver, spleen, lung, and kidney) and tumors were collected. Subsequently, the obtained tissues were weighted, cut into small pieces, and digested with *aqua regia* for three days. After that, each sample was diluted with water to have a volume of 10 mL and analyzed by ICP-OES to quantify the Au content in each sample (n = 3).

***In Vivo* Antitumor Therapeutic Efficacy:** The Pan02 tumor-bearing mice with a tumor volume of 100 mm^3 were randomly divided into 6 groups (n = 5 for each group), which were separately treated with PBS, Au@G3 NGs, free Toy, Au/Toy@G3 NGs, Au/Toy@G3 NGs + UTMD or Au/Toy@G3 NGs + UTMD + Anti-PD-L1 ([Toy] = 2 mg/kg for all Toy-related groups, and the concentration of Au@G3 NGs corresponded to the Toy-incorporated groups, [Anti-PD-L1] = 0.2 mg/mL). The mice were injected with the related materials *via* the tail vein every 2 days for 4 times. For the Au/Toy@G3 NGs + UTMD group, SonoVue (100 μL for each mouse) was intravenously injected right following the injection of NGs, and the UTMD was performed using a US transducer (DJO France SAS, Mouguerre, France) positioning above the tumor region (1 MHz, 0.4 W/cm^2, 20% MBs, and 2 min). For the Au/Toy@G3 NGs + UTMD + Anti-PD-L1 group, Anti-PD-L1 (0.2 mg/mL, 0.1 mL) was intratumorally administrated to each mouse on the other day after injection of Au/Toy@G3 NGs + UTMD. The tumor volume and body weight of each mouse were recorded every other day for 14 days, and the tumor volume was calculated as $V = a \times b^2 /2$ where a and b

represent the length and width of the tumor, respectively. The relative tumor volumes were calculated based on the tumor volume of mice before different treatments.

At the end of the treatments, US imaging was conducted using the transducer in both contrast-enhanced ultrasound (CEUS) mode and conventional B-mode at the same time points. US imaging was used to visualize the tumor size and blood perfusion using a clinical diagnostic ultrasound scanner, SIEMENS Healthineers Acuson sequoia (Siemens Shanghai Medical Equipment Ltd., Shanghai, China). In addition, mice after various treatments were injected with SonoVue MBs (1.18 mg/mL, 0.1 mL saline), and CEUS imaging was conducted using the same probe for the contrast mode at a low mechanical index (0.12). All CEUS images were collected under the same conditions (20 mm depth and 3 gains).

***In Vivo* Thermal Imaging of Tumors:** Pan02 tumor-bearing mice with a tumor volume of approximately 100 mm^3 were injected with Au/Toy@G3 NGs + UTMD ([Au] = 10 mM, in 100 μL PBS for each mouse) *via* the tail vein for thermal imaging. Before and after UTMD (0, 30 s, 1 min and 2 min), the whole body infrared thermography and the corresponding temperature values were obtained by a thermal imager (FLIR A300, IRS Systems Inc., Shanghai, China) according to our earlier study.[5]

Intratumoral Immune Cell Infiltration: Subsequently, to confirm the immunological effect induced by the treatments, the infiltration of immune cells in tumors was examined at 14 days post-treatment. The blood of the tumor-bearing mice was also collected from the orbital sinus (500 μL for each mouse), placed in the sterile centrifuge tube, stored at room temperature for 1 h, and then centrifuged at 416 g for 20 min to isolate the serum for evaluating immune-related cytokines including interferon-γ (IFN-γ) and TNF-α by ELISA (n = 3). Then, the tumor-bearing mice were sacrificed by cervical dislocation and immersed in 75% alcohol for 2-5 min, and the tumors in different groups were collected under sterile conditions and stored in icy PBS. To extract the tumor-infiltrating immune cells, the tumors were cut into small pieces and incubated with a digestion solution (1 mg/mL type IV

collagenase, 100 μg/mL DNase I, and 100 μg/mL hyaluronidase in DMEM) at 37 °C for 30 min with persistent agitation. Digested tumor samples were filtered by a 400-mesh sieve to obtain a single cell suspension, which was centrifuged at 104 g for 5 min and re-suspended in DMEM. Afterward, the lymphocyte suspension in the tumor tissue was separated according to the instructions of the tumor-infiltrating lymphocyte cell separation medium kit for mice. The prepared single-cell suspension of tumor-infiltrating tissue was mixed with an equal volume of lymphocyte separation solution, and centrifuged (500 g, 30 min) at room temperature. After centrifugation, the second lymphocyte layer was carefully separated, washed with cell washing solution, and collected to obtain the tumor-infiltrating lymphocytes. The lymphocytes were filtered by the nylon wool column to obtain the tumor-infiltrating T cells. Further, the T cells extracted from tumors were resuspended in PBS, stained with anti-CD4-FITC/anti-CD8-PE antibody or fixed with an intracellular staining kit (Thermo Fisher Scientific, Waltham, MA), and stained with anti-CD4-PE/anti-Foxp3-APC/anti-CD25-FITC (5 μL of each) antibodies before flow cytometry analysis (n = 3).

Histological Examinations: At 14 days post-treatment, each mouse in different groups was euthanized, and the major organs (heart, liver, spleen, lung, and kidney) were collected, fixed, and embedded in paraffin. The major organs were sectioned for hematoxylin and eosin (H&E) staining to observe the histological changes caused by different treatments according to the standard protocols.[4] The tumor tissues were sectioned into slices for TdT-mediated dUTP Nick-End Labeling (TUNEL), H&E, and Ki67 staining according to the literature.[6] The TUNEL and Ki67 staining images were applied to quantify the apoptosis and proliferation rate of tumor cells. The tumor tissue was also sectioned for CRT immunofluorescence staining, for immunofluorescence staining to analyze the expression of CD86 and CD206 to evaluate the macrophage polarization in the TME, and for XBP1s and pIRE1α immunofluorescence staining and CHOP immunohistochemistry staining.[7] The spleens were also sectioned for CD4 and CD8 immunofluorescence staining to analyze the

activation of the immune system.

Biosafety Examinations: To verify the biosafety of different treatments after injection, healthy C57BL/6 mice (5-6 weeks old) were randomly divided into three groups (n = 3 in each group) and administrated with PBS, free Toy, and Au/Toy@G3 NGs according to the above protocols used for tumor treatment. At 7 days post-injection, the mice were euthanized and the blood was collected and stabilized with heparin for blood routine and blood biochemical analysis. The blood cell counts were performed on an automated blood cell counter (BC-2800 Vet Analyzers, Mindray, Shenzhen, China) for blood routine analysis, including red blood cell (RBC), platelet (PLT), white blood cell (WBC), and lymphocyte (Lymph). Then the blood samples were collected in the sterile centrifuge tubes, maintained at room temperature for 1 h, and then centrifuged at 416 g for 20 min to obtain the serum, and the serum biochemistry markers including alanine aminotransferase (ALT), aspartate aminotransferase (AST), creatinine (CR), and uric acid (UA) were analyzed by Servicebio Technology Co., Ltd. (Wuhan, China).

Statistical Analysis: All experimental data were represented as the mean ± standard deviation through at least three parallel experiments. One-way analysis of variance statistical method was used to analyze the experimental results through IBM SPSS Statistic 25 software (IBM, Armonk, NY). A p value of 0.05 was selected as a significance level, and the data were indicated with (*) for $p < 0.05$, (**) for $p < 0.01$, and (***) for $p < 0.001$, respectively.

Table S1. EE and LC of Toy in the Au/Toy@G3 NGs at different G3.NH_2/Toy feeding mass ratios.

G3.NH_2/Toy mass ratio	EE (%)	LC (%)
1: 0.1	66.7	6.3
1: 0.25	57.0	12.5
1: 0.5	37.0	15.6

Table S2. Hydrodynamic size and zeta potentials of different samples.

Sample	Hydrodynamic size (nm)	Zeta potential (mV)	Polydispersity index
G3 NGs	237.9 ± 6.4	32.8 ± 0.5	0.326 ± 0.142
G3 NGs（+GSH）	125.8 ± 2.9	17.1 ± 1.0	0.233 ± 0.079
Au@G3 NGs	204.7 ± 3.1	9.1 ± 0.1	0.306 ± 0.046
Au/Toy@G3 NGs	193.0 ± 4.1	2.9 ± 0.4	0.360 ± 0.058

Table S3. The IC_{50s} of free Toy, Toy + UTMD, Au/Toy@G3 NGs, and Au/Toy@G3 NGs + UTMD towards Pan02 cells after 24 h treatment.

Sample	IC_{50} (μg/mL)
Toy	5.06
Toy + UTMD	3.43
Au/Toy@G3 NGs	2.32
Au/Toy@G3 NGs + UTMD	1.59

Table S4. Primers are used to detect the mRNA expression of the ERS-related genes.

Gene	Oligo name	Sequence 5'-3'
GRP78	GRP78-F	TCATCGGACGCACTTGGAA
	GRP78-R	CAACCACCTTGAATGGCAAGA
XBP1u	XBP1u-F	GACAGAGAGTCAAACTAACGTGG
	XBP1u-R	GTCCAGCAGGCAAGAAGGT
XBP1s	XBP1s-F	AAGAACACGCTTGGGAATGG
	XBP1s-R	CTGCACCTGCTGCGGAC
CHOP	CHOP-F	GTCCCTAGCTTGGCTGACAGA
	CHOP-R	TGGAGAGCGAGGGCTTTG
β-actin	β-actin-F	AGAGGGAAATCGTGCGTGAC
	β-actin-R	GCGTCCACGTAGTAGTAGCC

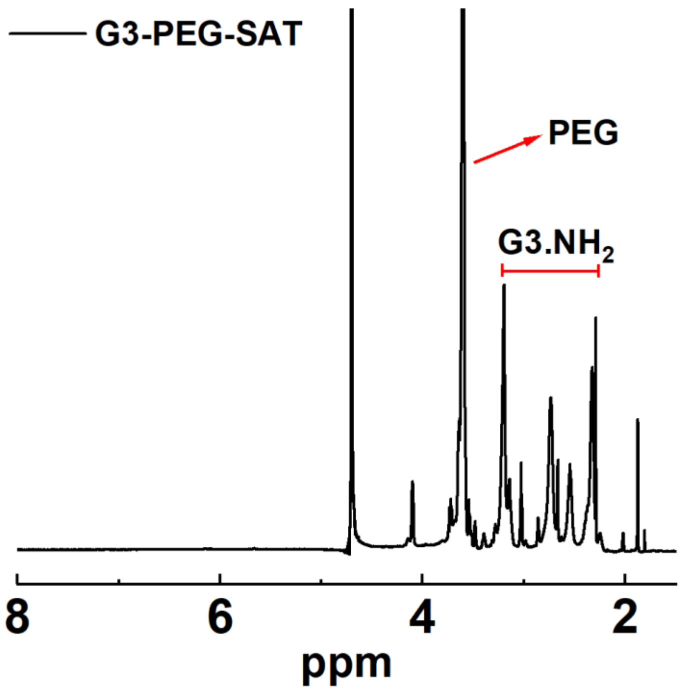

Figure S1. ^{1}H NMR spectrum of G3-PEG-SAT.

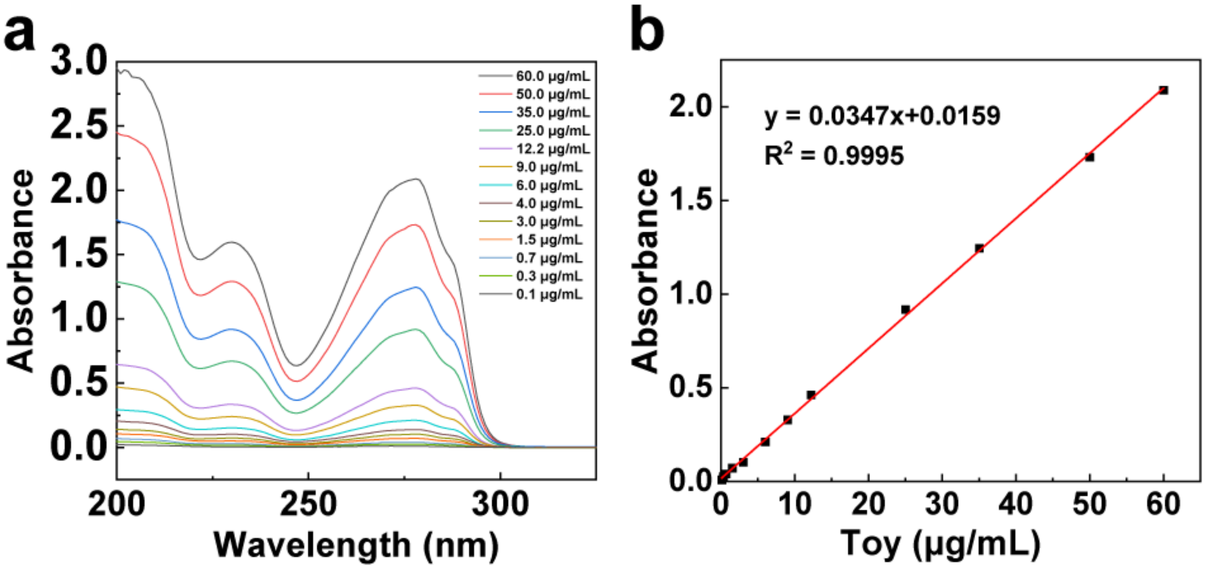

Figure S2. (a) UV-vis spectra of free Toy dissvoled in water at different concentrations. (b) Toy absorbance at 280 nm/concentration calibration curve.

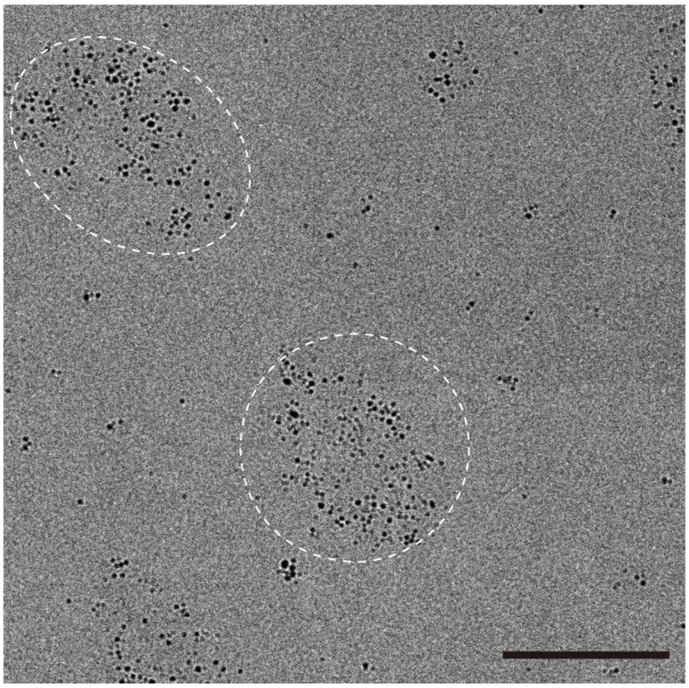

Figure S3 TEM image of Au@G3 NGs (scale bar = 200 nm).

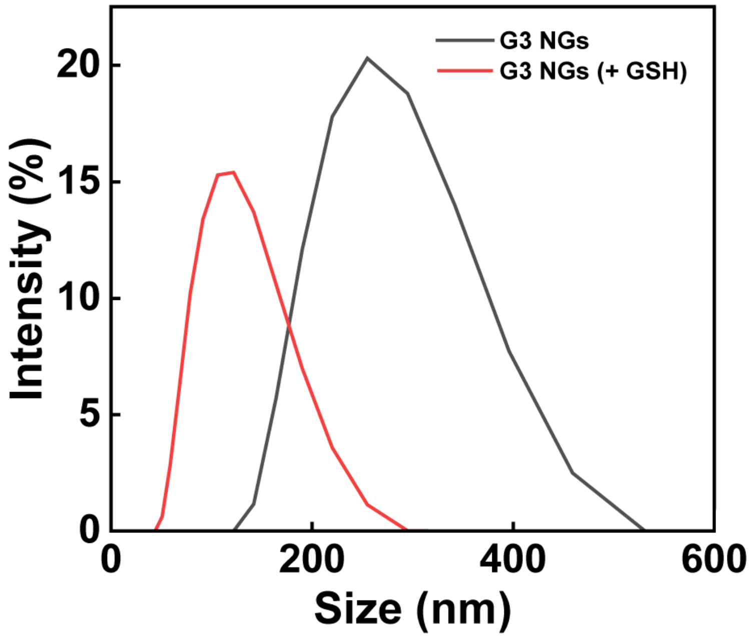

Figure S4. The hydrodynamic size distribution of G3 NGs with or without GSH (10 mM).

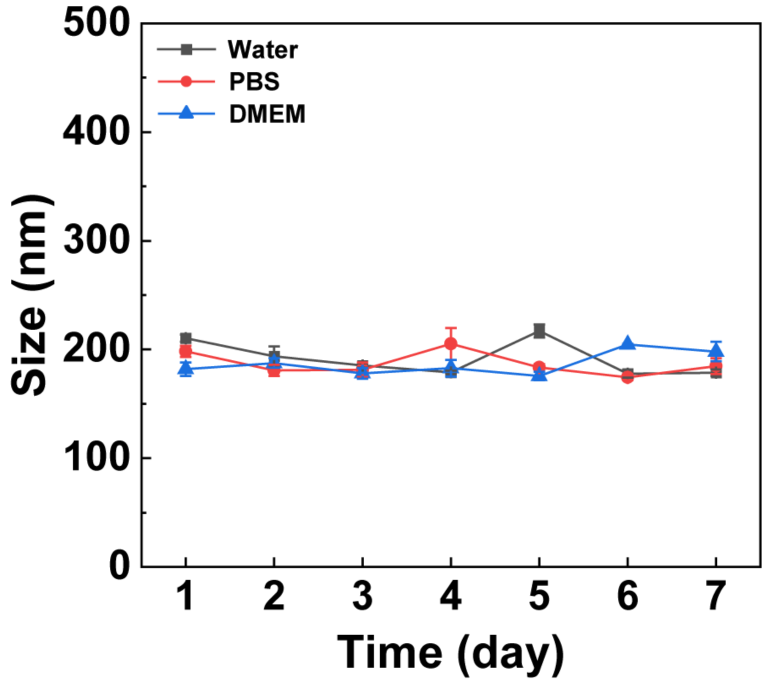

Figure S5. The hydrodynamic size of the Au@G3 NGs dispersed in water, PBS or DMEM (containing 10% FBS) for one week at room temperature (n = 3, and data are presented as mean ± SD).

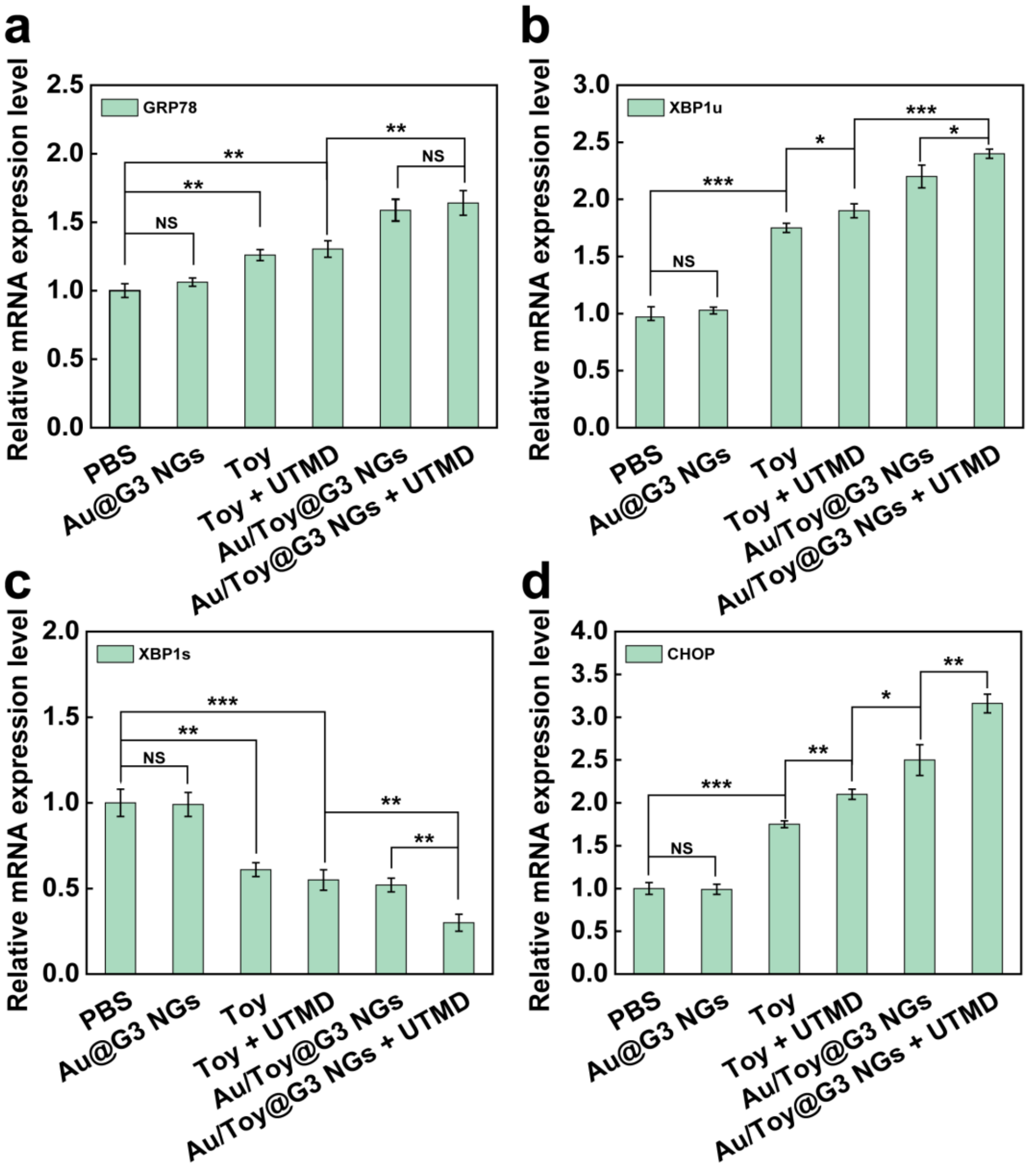

Figure S6. RT-PCR analysis of mRNA expression levels of ERS-related factors of (a) GRP78, (b) XBP1u, (c) XBP1s, and (d) CHOP in Pan02 cells treated with PBS, Au@G3 NGs, Toy, Toy + UTMD, Au/Toy@G3 NGs, and Au/Toy@G3 NGs + UTMD for 6 h, respectively (n = 3, data are presented as mean ± SD, and * is for $p < 0.05$, ** is for $p < 0.01$, and *** is for $p < 0.001$, respectively).

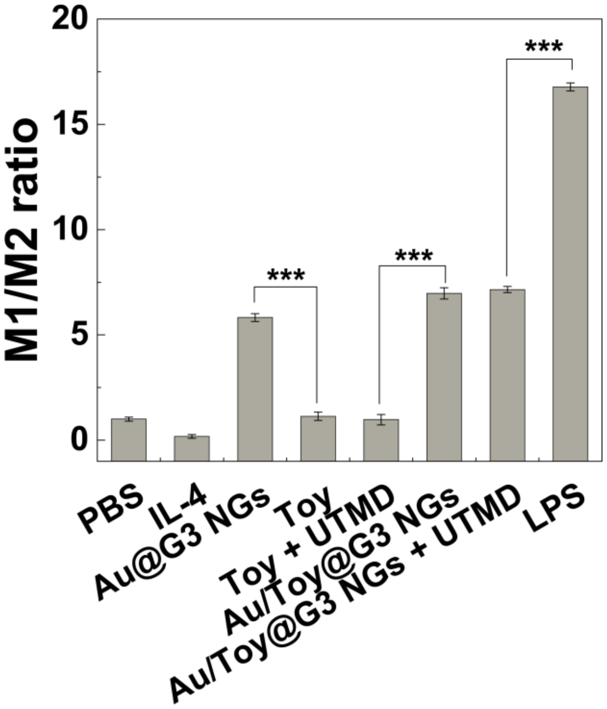

Figure S7. The quantification of the M1/M2 ratio in RAW264.7 cells treated with PBS, Au@G3 NGs, Toy, Toy + UTMD, Au/Toy@G3 NGs, Au/Toy@G3 NGs + UTMD or LPS (the RAW264.7 cells were pre-treated with IL-4 to form M2-type macrophages, n = 3, and data are presented as mean ± SD). Here, *** is for $p < 0.001$.

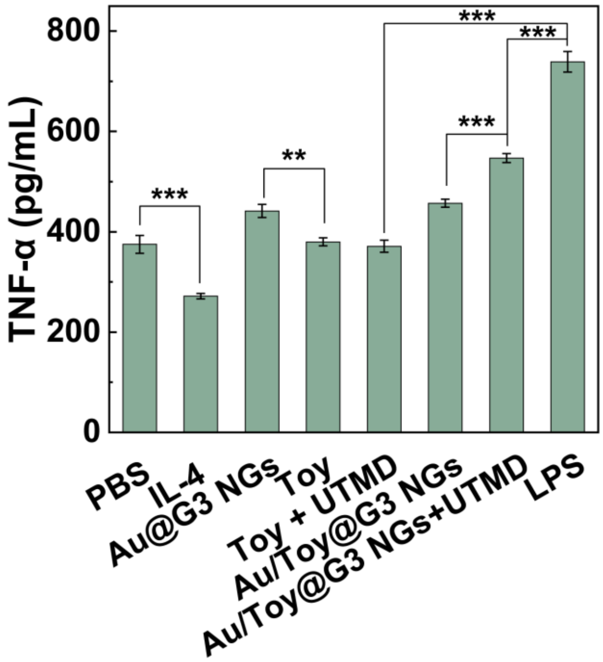

Figure S8. The secretion of TNF-α in the culture medium of RAW264.7 cells after different treatments (n = 3, data are presented as mean ± SD, and ** is for $p < 0.01$, and *** is for $p < 0.001$, respectively).

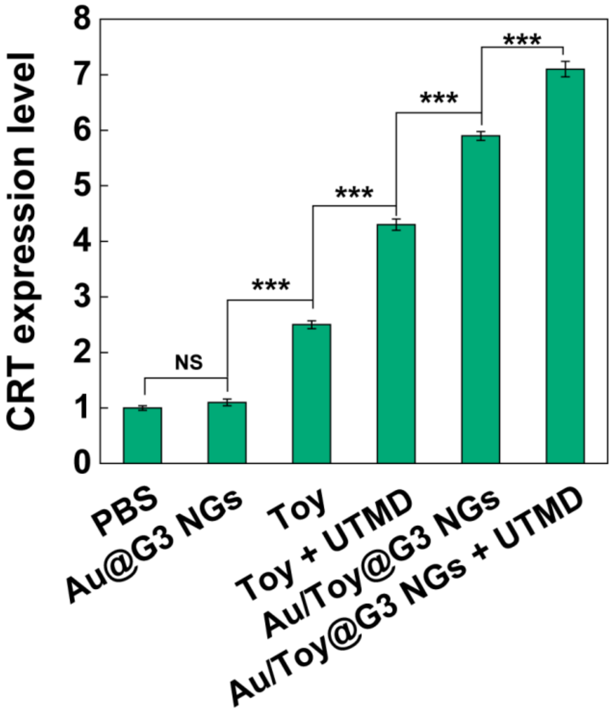

Figure S9. Quantitative analysis of CRT expression in pan02 cells after different treatments (n = 3, data are presented as mean ± SD, and *** is for p < 0.001).

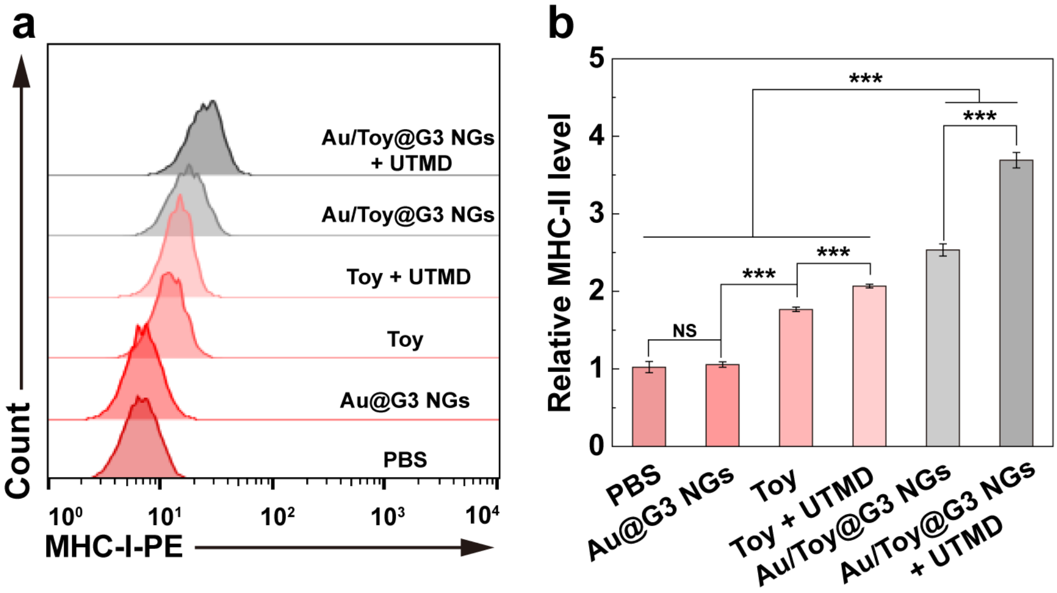

Figure S10. Flow cytometric analysis of MHC-I expression on DCs after co-culture with ICD Pan02 cells that were differently treated: (a) flow cytometric histograms and (b) quantitative analysis of MHC-I expression. In part B, data are presented as mean ± SD (n = 3), and NS is for no significant difference, and *** is for p < 0.001, respectively.

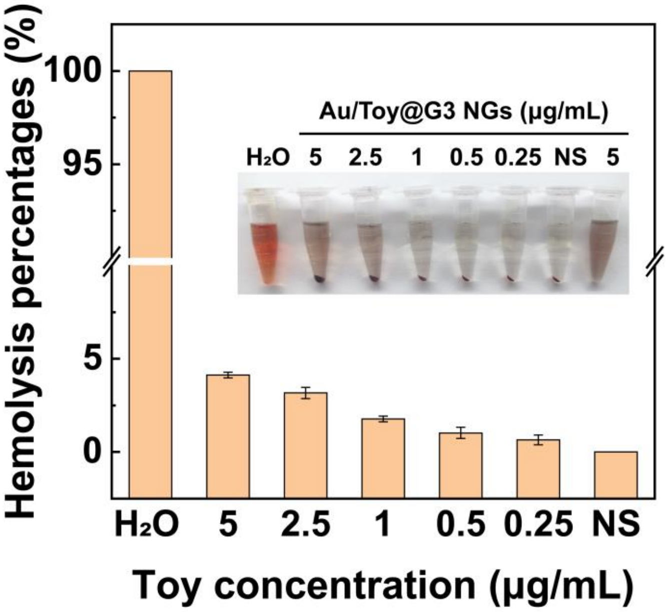

Figure S11. Hemolysis percentages of mouse RBCs treated with the Au/Toy@G3 NGs at different Toy concentrations for 2 h (n = 3, data are presented as mean ± SD). Inset shows the corresponding digital photos of the RBC suspensions after 2 h incubation followed by centrifugation and the right most centrifuge tube is Au/Toy@G3 NGs (5 μg/mL) with their own color. Water and NS were used as positive and negative controls, respectively.

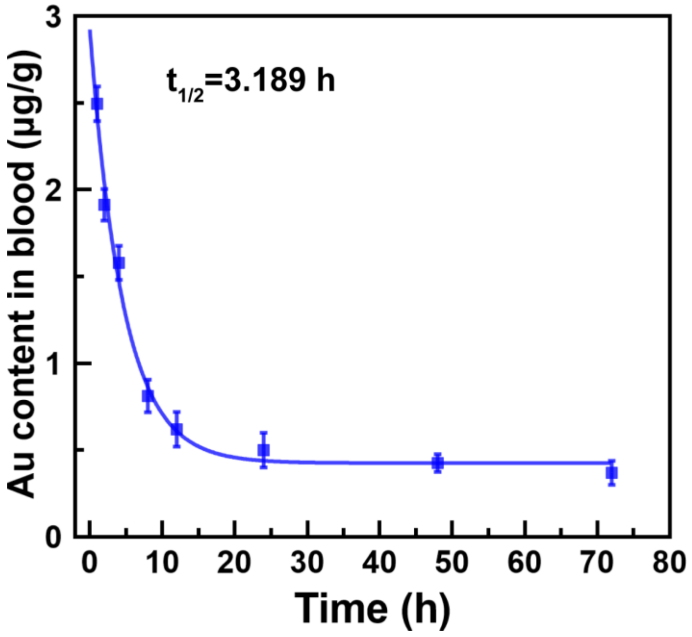

Figure S12. The pharmacokinetics of Au/Toy@G3 NGs after i.v. injection to mice ([Au] = 4 mM, 100 μL, n = 3, data are presented as mean ± SD).

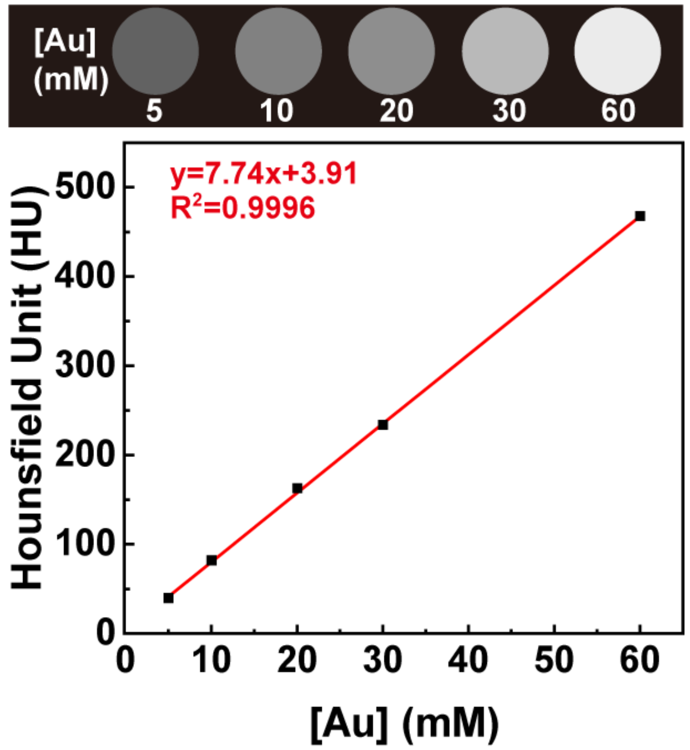

Figure S13. *In vitro* CT images and CT values of Au/Toy@G3 NGs at different Au concentrations.

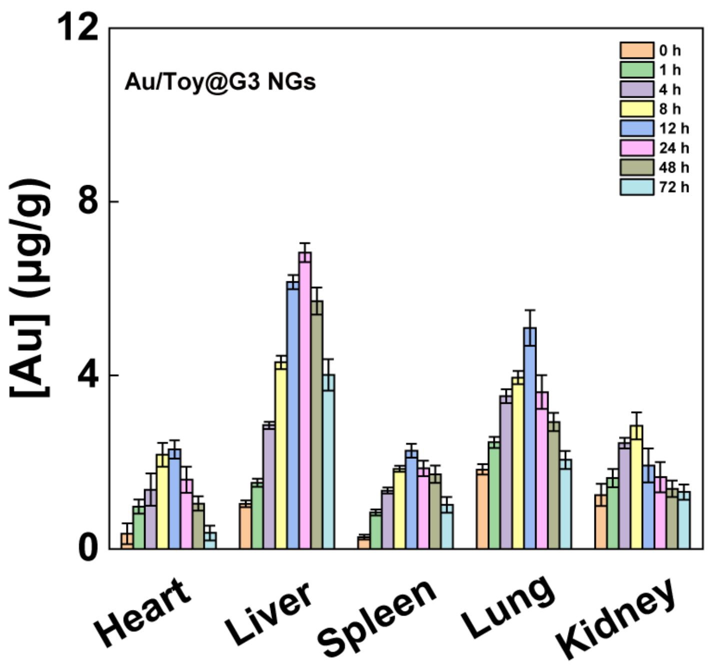

Figure S14. The biodistribution of Au in major organs of mice at different time points post intravenous injection of Au/Toy@G3 NGs ([Au] = 10 mM, 0.1 mL, n = 3, data are presented as mean ± SD).

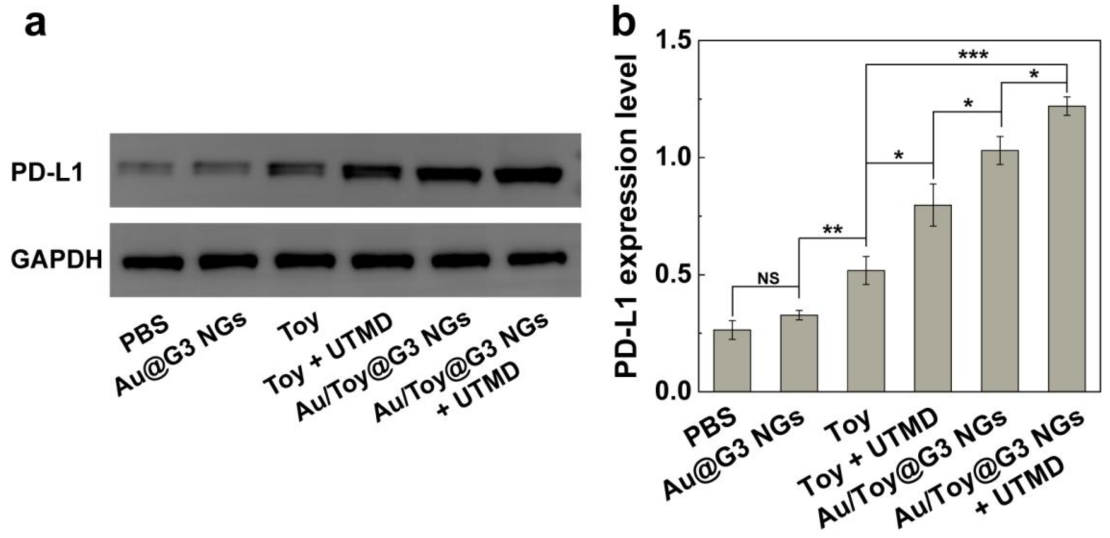

Figure S15. (a) WB analysis and (b) the quantification of the expression levels of PD-L1 on Pan02 cells after incubation with PBS, Au@G3 NGs, Toy, Toy + UTMD, Au/Toy@G3 NGs or Au/Toy@G3 NGs + UTMD for 24 h. In part b, data are presented as mean ± SD (n = 3), and NS is for no significant difference, * is for $p < 0.05$, ** is for $p < 0.01$, and *** is for $p < 0.001$, respectively.

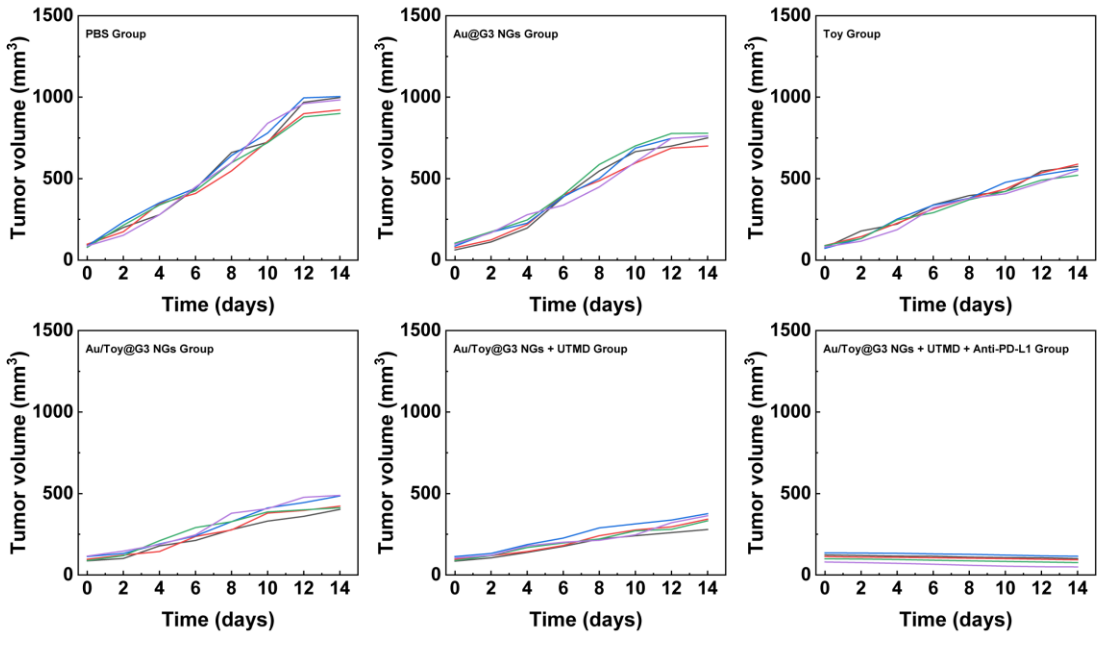

Figure S16. Tumor volume changes of mice after different treatments (n = 5).

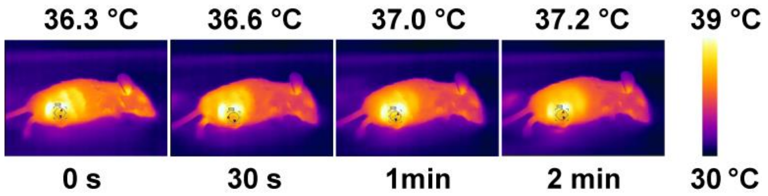

Figure S17. Infrared thermal images of tumors before (0 min) and after UTMD at different time periods.

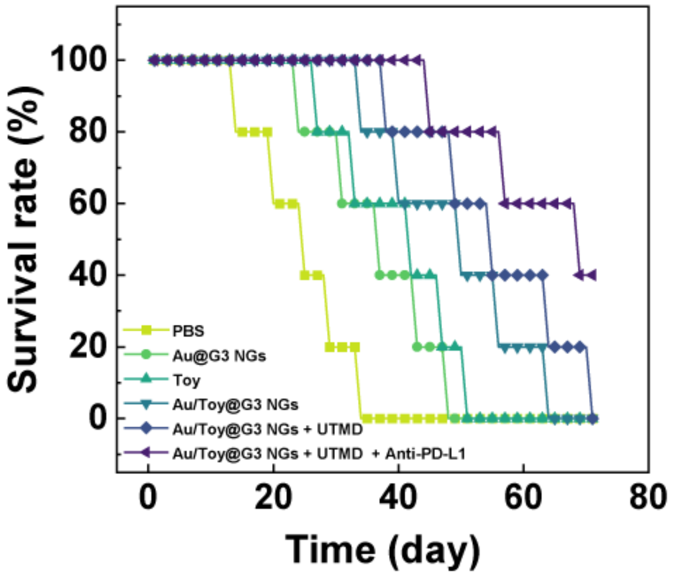

Figure S18. Survival rate of Pan02 tumor-bearing mice after different treatments (n = 5 for each group).

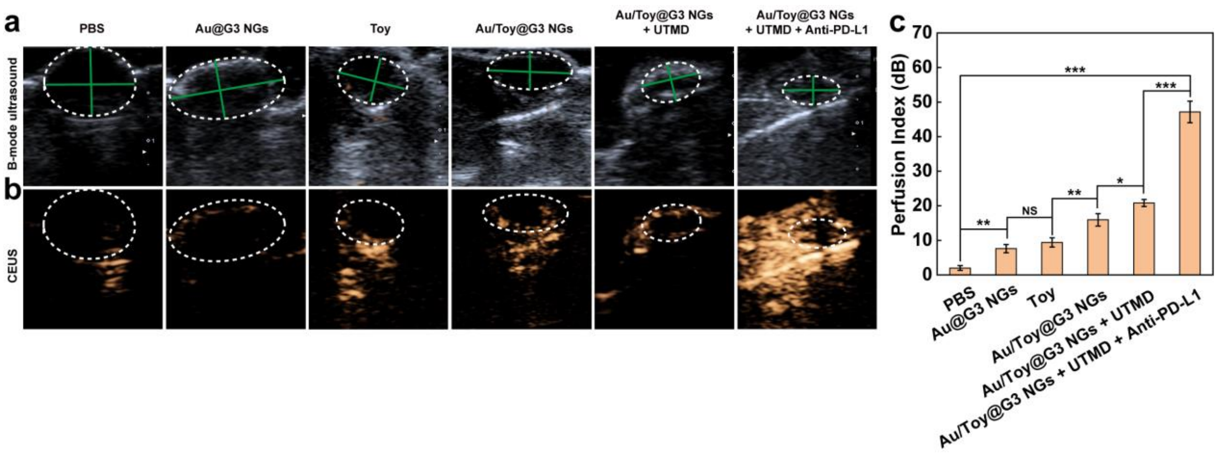

Figure S19. (a) Two-dimensional gray scale ultrasound images, (b) CEUS images, and (c) intratumoral perfusion index of the mouse in different groups. The white ovals represent the tumor site (n = 3, data are presented as mean ± SD, and * is for $p < 0.05$, ** is for $p < 0.01$, and *** is for $p < 0.001$, respectively).

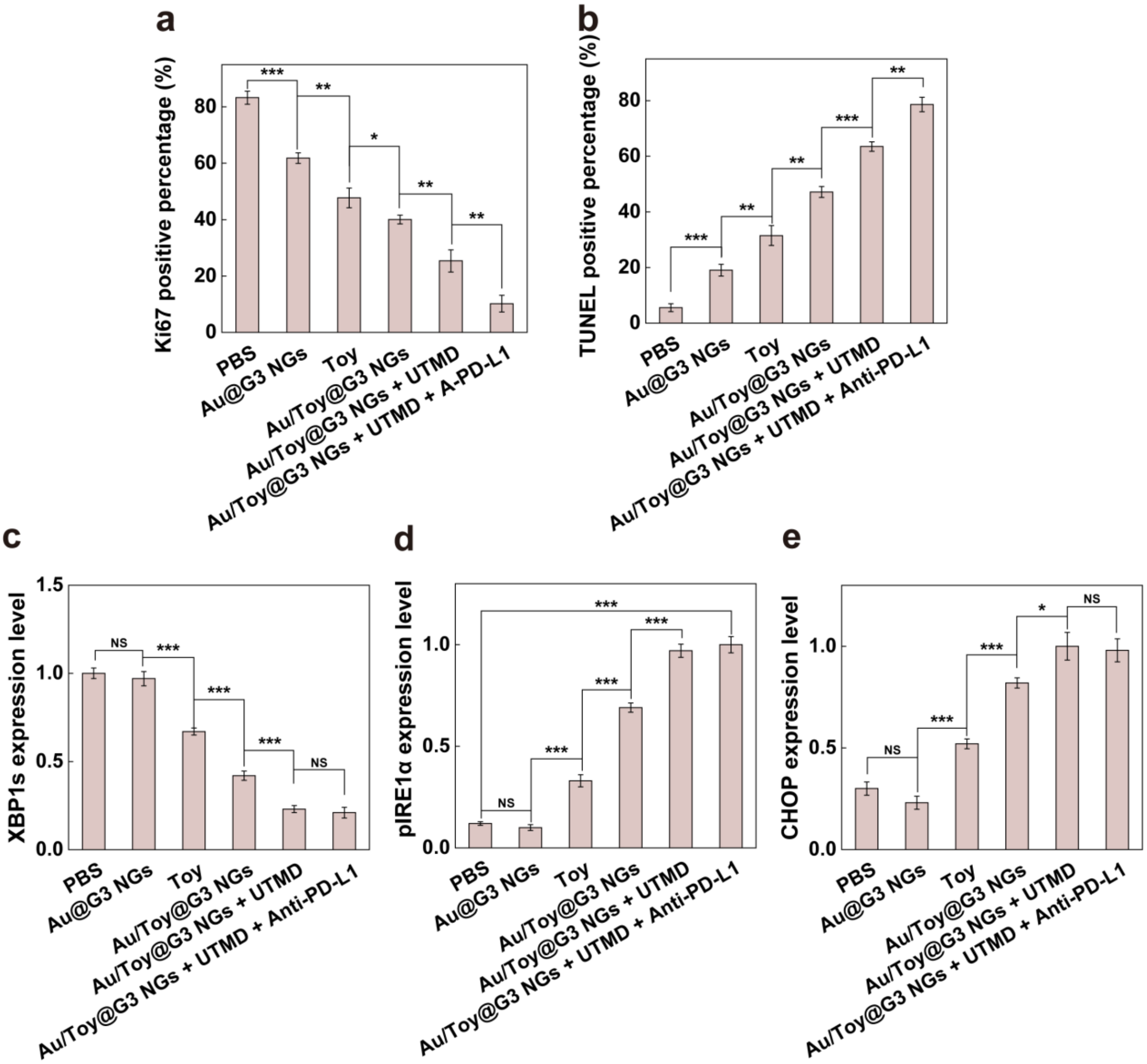

Figure S20. Quantitative analysis of (a) Ki67-positive cells, (b) TUNEL-positive cells, (c) XBP1s expression level, (d) pIRE1α expression level, and (e) CHOP expression level in tumor tissue after different treatments ($n = 3$, data are presented as mean ± SD, and * is for $p < 0.05$, ** is for $p < 0.01$, and *** is for $p < 0.001$, respectively).

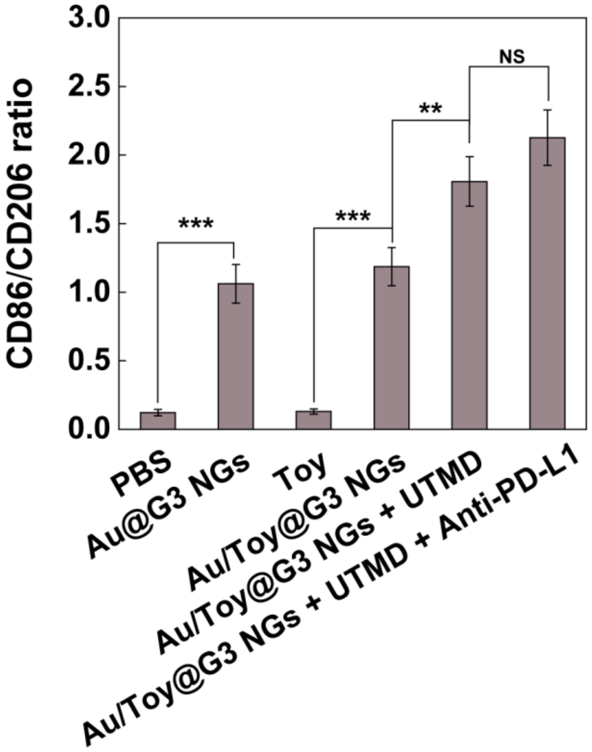

Figure S21. Quantitative analysis of CD86/CD206 ratio in tumor sections after different treatments (n = 3, data are presented as mean ± SD, ** is for p < 0.01, and *** is for p < 0.001, respectively).

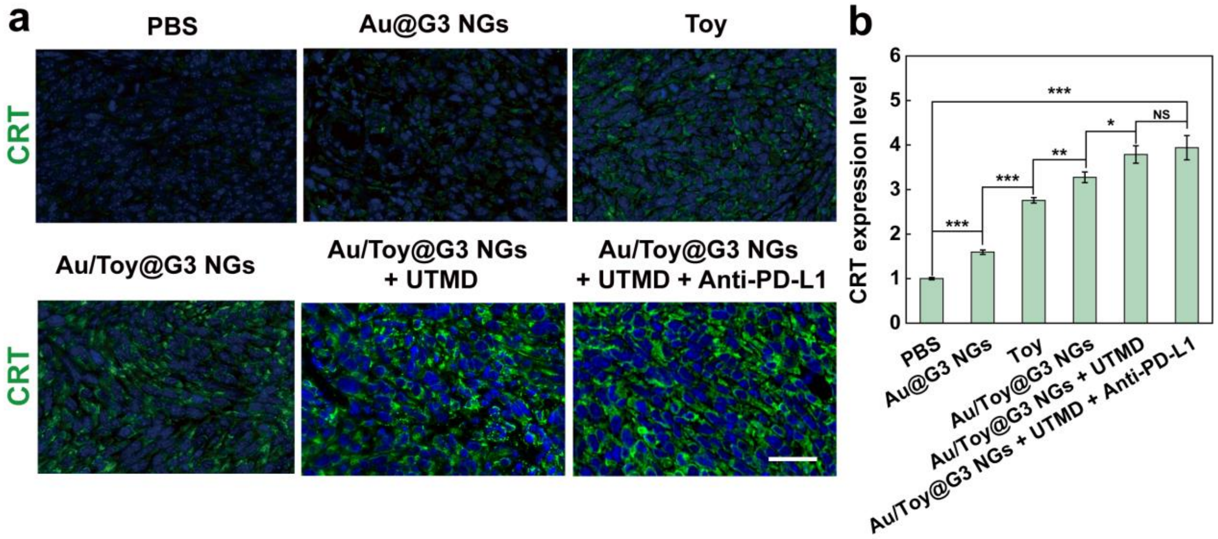

Figure S22. (a) The immunofluorescence staining of CRT and (b) quantitative analysis of CRT expression in tumor sections after the mice were treated for 14 days in different groups (n = 3). The scale bar for each panel represents 50 μm. For (b), data are presented as mean ± SD, and * is for p < 0.05, ** is for p < 0.01, and *** is for p < 0.001, respectively.

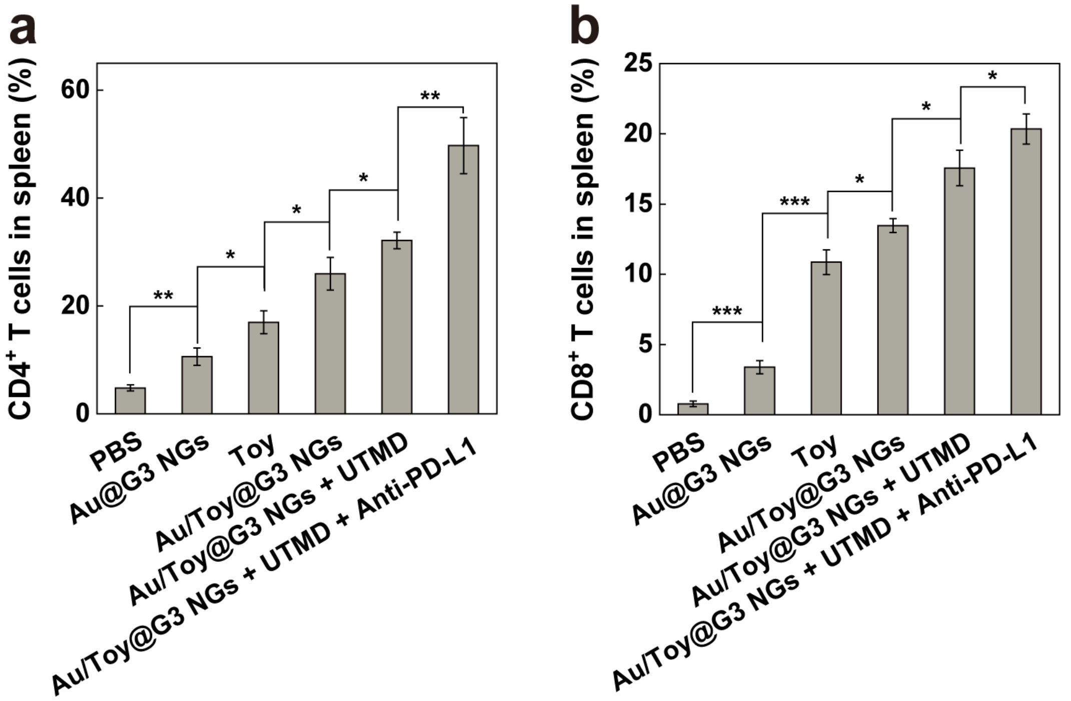

Figure S23. Quantitative analysis of (a) CD4$^+$ T cells and (b) CD8$^+$ T cells in spleens after the mice were treated for 14 days in different groups (n = 3, data are presented as mean ± SD, and * is for p < 0.05, ** is for p < 0.01, and *** is for p < 0.001, respectively).

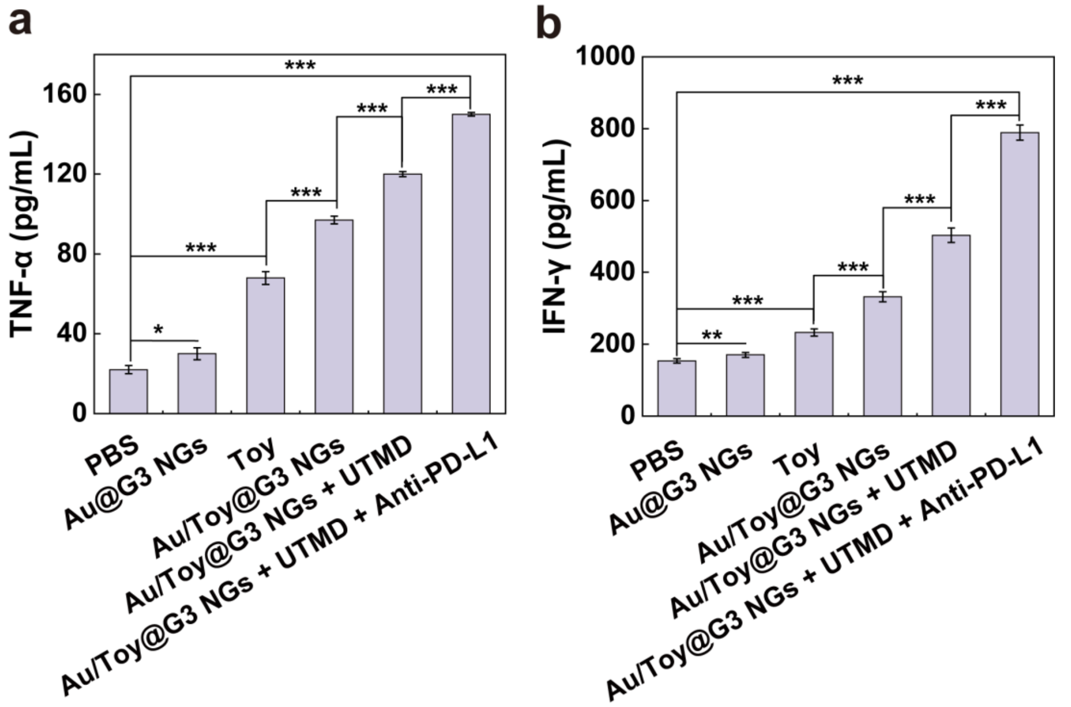

Figure S24. The levels of (a) TNF-α and (b) IFN-γ in serum after 14 days treatment in different groups as measured by ELISA kit (n = 3, data are presented as mean ± SD, and * is for p < 0.05, ** is for p < 0.01, and *** is for p < 0.001, respectively).

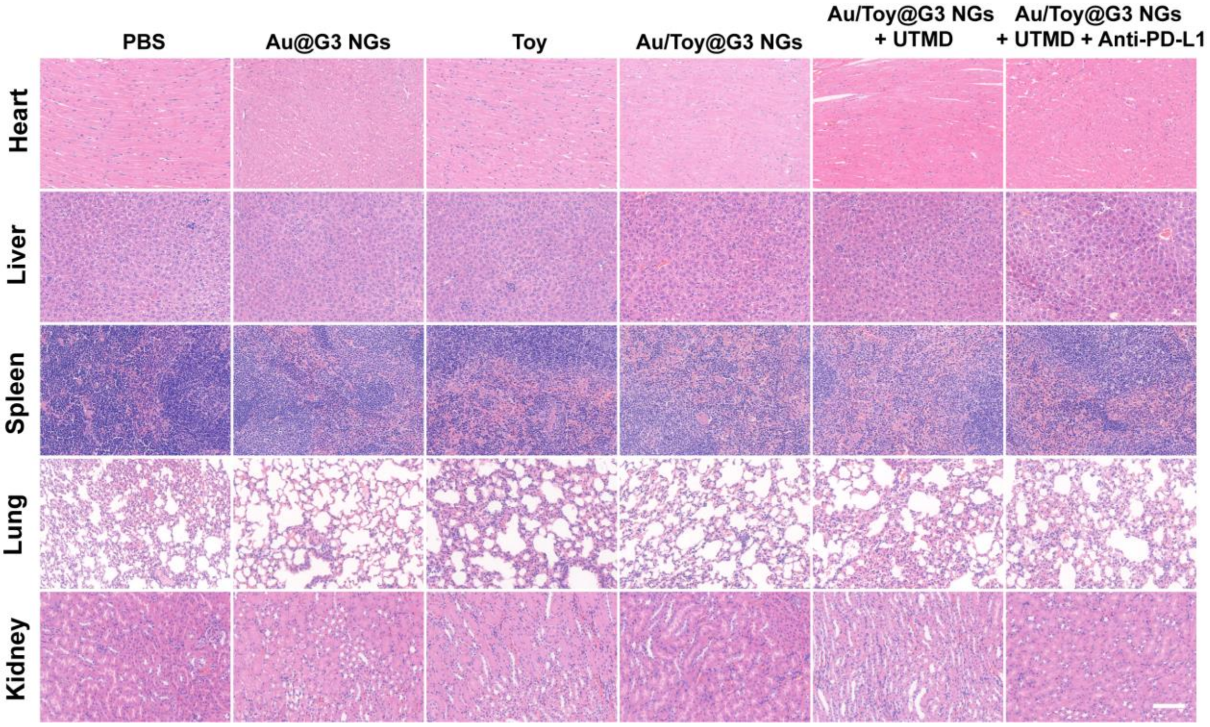

Figure S25. Representative H&E-stained sections of major organs including heart, liver, spleen, lung, and kidney of tumor-bearing mice after different treatments for 14 days. The scale bar for each panel represents 100 μm.

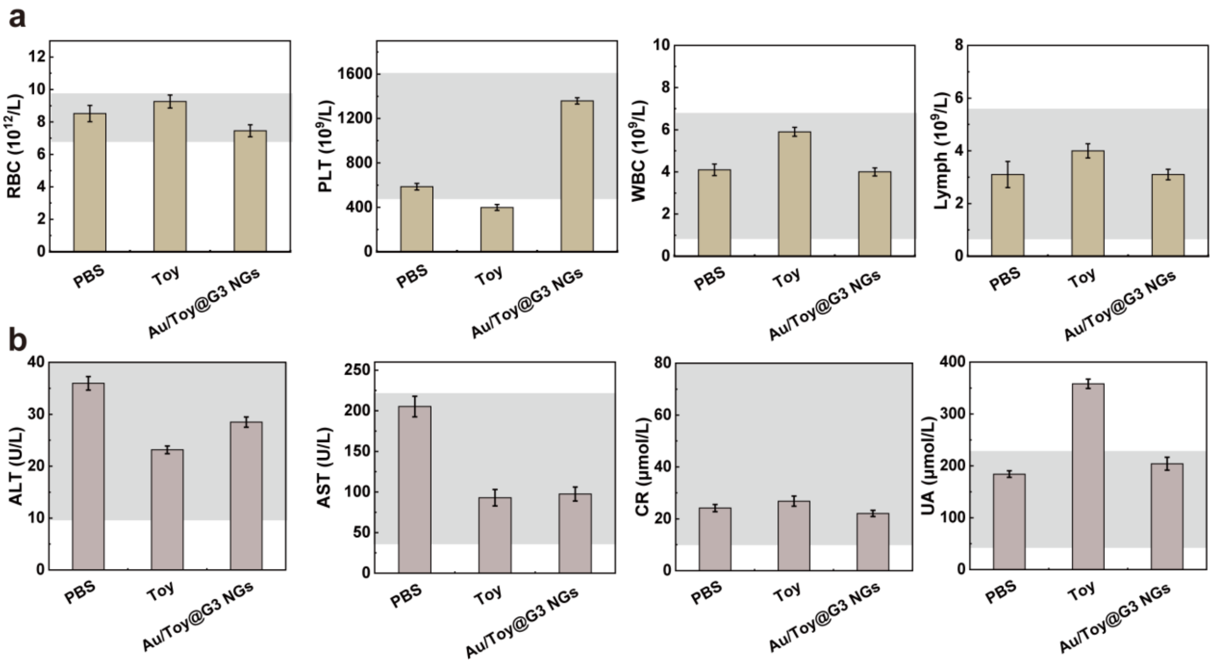

Figure S26. (a) The blood routines analysis including levels of red blood cells (RBC), platelet (PLT), white blood cells (WBC), and lymphocytes (Lymph) of healthy nude mice after being treated with PBS, Toy or Au/Toy@G3 NGs for 7 days. (b) Blood biochemistry data including liver function markers (alanine aminotransferase (ALT), and aspartate aminotransferase (AST)), and kidney function markers (creatinine (CR) and uric acid (UA)) of healthy nude

mice after treatments with PBS, Toy or Au/Toy@G3 NGs for 7 days. PBS-treated healthy nude mice were used as control (n = 3 for each sample, and data are presented as mean ± SD). The grey area represents the normal range of the corresponding parameter.

References

[1] L. Lin, Y. Fan, F. Gao, L. Jin, D. Li, W. Sun, F. Li, P. Qin, Q. Shi, X. Shi, L. Du, *Theranostics* **2018**, 8, 1923.

[2] Y. Fan, L. Lin, F. Yin, Y. Zhu, M. Shen, H. Wang, L. Du, S. Mignani, J.-P. Majoral, X. Shi, *Nano Today* **2020**, 33, 100899.

[3] Y. Guo, Y. Fan, Z. Wang, G. Li, M. Zhan, J. Gong, J. P. Majoral, X. Shi, M. Shen, *Adv. Mater.* **2022**, 34, 2206861.

[4] Y. Xu, Y. Guo, C. Zhang, M. Zhan, L. Jia, S. Song, C. Jiang, M. Shen, X. Shi, *ACS Nano* **2022**, 16, 984.

[5] C. Zhang, W. Sun, Y. Wang, F. Xu, J. Qu, J. Xia, M. Shen, X. Shi, *ACS Appl. Mater. Interfaces* **2020**, 12, 9107.

[6] G. M. Li, Y. Fan, L. Z. Lin, R. Wu, M. W. Shen, X. Y. Shi, *Sci. China: Chem.* **2021**, 64, 817.

[7] a) M. Fan, S. Chen, Y. Weng, X. Li, Y. Jiang, X. Wang, M. Bie, L. An, M. Zhang, B. Chen, G. Huang, J. Wu, M. Zhu, Q. Shi, *Oncol. Rep.* **2020**, 44, 91; b) H. Fukushima, S. Yoshida, T. Kijima, Y. Nakamura, S. Fukuda, S. Uehara, Y. Yasuda, H. Tanaka, M. Yokoyama, Y. Matsuoka, Y. Fujii, *Int. J. Mol. Sci.* **2021**, 22, 535; c) Z. Wang, Y. Guo, Y. Fan, J. Chen, H. Wang, M. Shen, X. Shi, *Adv. Mater.* **2021**, 34, 2107009.
